# Long-term stability and performance of Cas9/guide RNA-based gene drives in anopheline mosquitoes

**DOI:** 10.1073/pnas.2605739123

**Published:** 2026-07-06

**Authors:** Rebeca Carballar-Lejarazú, Yuemei Dong, Thai Binh Pham, Taylor Tushar, Drusilla Stillinger, Devin Ngoc Nguyen, Lorena Winokur, Mihra Tavadia, Mabel Tao, George Dimopoulos, Anthony A. James

**Affiliations:** ^a^ Department of Microbiology and Molecular Genetics, University of California, Irvine, CA 92697-4025; ^b^ W. Harry Feinstone Department of Molecular Microbiology and Immunology, Bloomberg School of Public Health, Malaria Research Institute, Johns Hopkins University, Baltimore, MD 21205; ^c^ Department of Molecular Biology and Biochemistry, University of California, Irvine, CA 92697-3900

**Keywords:** CRISPR, single chain antibodies, antimicrobial peptides, target product profile, Plasmodium falciparum

## Abstract

Gene-drive population modification strategies are being developed to control the transmission by anopheline mosquitoes of the parasites that cause human malaria. These approaches are designed to reduce disease prevalence and incidence by spreading dominant antiparasite effector genes throughout vector populations. The strains must sustain drive and parasite suppression properties over extended periods of time to have an epidemiological impact. Three gene-drive strains, AcTP13 and AcTP43 in *Anopheles coluzzii* and AgTP13 in *Anopheles gambiae*, carrying autonomous Cas9/guide RNA-based drive systems linked to multiple antiparasite effector genes were remarkably stable in all *A. coluzzii* replicates over a 2-y (35 generation) period in laboratory cage trials. Two of three *A. gambiae* replicates performed equally well. Stability was assessed as a function of population dynamics (size), molecular integrity of the gene-drive cassettes, maintenance of drive efficiency (gene conversion), generation and accumulation of mutant drive-resistant target alleles, drive system-generated off-target effects, and effector gene parasite suppression activity. All lines met stability requirements with the exception of one AgTP13 cage replicate that was affected by drive-resistant target-site mutations. Notably, all strains retained parasite suppression activity and high drive efficiencies throughout the duration of the trials. These results support the further development and deployment of these strains for malaria control.

## Significance

Gene-drive systems for population modification of mosquitoes that transmit human malaria parasites have promise as sustainable approaches for malaria control. The impact of these systems on disease prevalence and incidence requires long-term stability of both the drive mechanisms and the effector genes that suppress the parasites. We demonstrate the stability of two drive systems, TP13 and TP43, in the African malaria mosquitoes, *Anopheles coluzzii* and *Anopheles gambiae* over a 2-y, 35 generation period. The robust linkage between the drive system dynamics and the impact on parasite suppression bridges a key translational gap between gene-drive mechanics and malaria control relevance. This work provides a comprehensive durability benchmark that can inform risk assessment and the design of next-generation multieffector, resistance-resilient drive systems.

Malaria remains a major world-wide health concern and is caused by *Plasmodium* parasites transmitted to humans by mosquitoes in the genus *Anopheles*. Despite recent reductions in mortality, disease incidence is on the rise (1). The impacts of once-effective control strategies, including insecticide applications and drug therapies, are threatened by increasing insecticide and drug resistance, inconsistent implementation, and limited sustainability (2). Consequently, genetic technologies, and specifically CRISPR-based gene drives, have gained significant attention as potential transformative malaria control tools (3).

Gene-drive systems operate by biasing inheritance patterns of specific genes and enable the rapid spread of desirable traits throughout target populations. Population modification, which introduces genetic elements that block *Plasmodium* transmission, represents a potentially stable and efficient approach to malaria control (4, 5). Laboratory studies have demonstrated efficient propagation of Cas9/guide RNA (gRNA)-based systems in the African malaria vectors, *Anopheles gambiae* and *A. coluzzii*, with epidemiologically significant expression of antiparasitic effector genes and reductions in parasite infection prevalence and intensities (6, 7).

Gene drives must meet Target Product Profile (TPP) objectives to transition from laboratory studies to field implementation and these set the minimally acceptable performance necessary for safe, effective, and scalable deployment. Gene-drive TPP criteria include high transmission efficiencies (inheritance biases >90% to spread beneficial genes rapidly through vector populations), versatility (active in diverse genetic backgrounds and ecological conditions without loss of function or drive breakdown), minimal fitness costs (negligible genetic loads to avoid selective disadvantages that compromise spread), low drive resistance (minimizing the generation and selection of cleavage-resistant alleles that suppress drive function), containment and monitoring (methods for molecular monitoring, resistance detection, and potential mitigation) and operational feasibility (compatibility with existing vector control programs and regulatory frameworks, including scalability for mass rearing and release) (3).

Many TPP parameters have been met successfully in laboratory studies of gene-drive strains, but questions remain regarding their long-term stability under conditions of ecological complexity and genetic heterogeneity (6–9). Factors such as gene target site variability (polymorphisms), resistance allele formation, loss of gene function, off-target effects, and unintended fitness effects could challenge the durability of these systems. While modeling studies have explored some of these risks, empirical evidence from extended multigenerational studies is needed (10, 11).

We present here the results of a multimetric, multireplicate, multiyear investigation of the long-term stability of Cas9/gRNA-based gene drives designed for population modification of *Anopheles* mosquitoes. Three gene-drive lines in two species, AcTP13 and AcTP43 in *A. coluzzii* and AgTP13 in *A. gambiae*, carrying autonomous drive systems along with dual or multieffector anti-*Plasmodium* genes, were shown to have high drive efficiencies, significant parasite blocking activity, good fitness based on life-table analyses and rapid fixation in short-term small cage trials (6, 7). We assess here drive inheritance dynamics, molecular fidelity, and phenotypic persistence over 2 y (35 generations) in laboratory cage trials and provide essential insights into the durability of gene drives relative to the TPP framework. Our findings inform risk assessment, product development, and strategic planning for future applications of these technologies in malaria-endemic regions.

## Results

The TP13 and TP43 gene-drive systems target the 2nd chromosome *cardinal* gene (*Agcd*) ortholog and detailed descriptions of the phenotypes, genotypes, and gene-drive terminology have been reported previously (9, 12). Expected visible phenotypes include “black” eyes conferred by the dominant wild-type allele, *cd*^+^, recessive “red” eyes resulting from homozygous nonfunctional mutant alleles, *cd*^−^, blue-fluorescent eye phenotypes resulting from the expression of the dominant marker gene encoding the Cyan Fluorescent Protein (CFP^+^) and red-fluorescent body phenotypes resulting from mCherry (mCherry^+^) expression. Fluorescent phenotypes mark the presence of a gene-drive system in the mosquitoes. An exceptional “tear” eye phenotype is a mosaic of red (mCherry^+^) or blue (CFP^+^) and black ommatidia visible in pupal and adult eyes resulting from paternally- or maternally deposited Cas9/gRNA complexes acting on target genes in somatic cells during development (6, 8). Phenotypes not accountable by Mendelian segregation patterns also are deemed exceptional. A “target” allele is any wild-type *cd*^+^ allele potentially subject to Cas9/gRNA-mediated cleavage.

### Population Dynamics.

Drive-system stability was assessed using discrete generation (nonoverlapping) competitive cage trials initiated with a 1:1, gene drive:wild-type (WT) male release ratio (13). Triplicate cages were seeded with 75 homozygous gene-drive males (AgTP13, AcTP13, or AcTP43), 75 wild-type (WT-X1 for AgTP13, and WT-Mopti for AcTP13 and AcTP43) males, and 150 each of the corresponding WT virgin females.

**Fig. 1.**
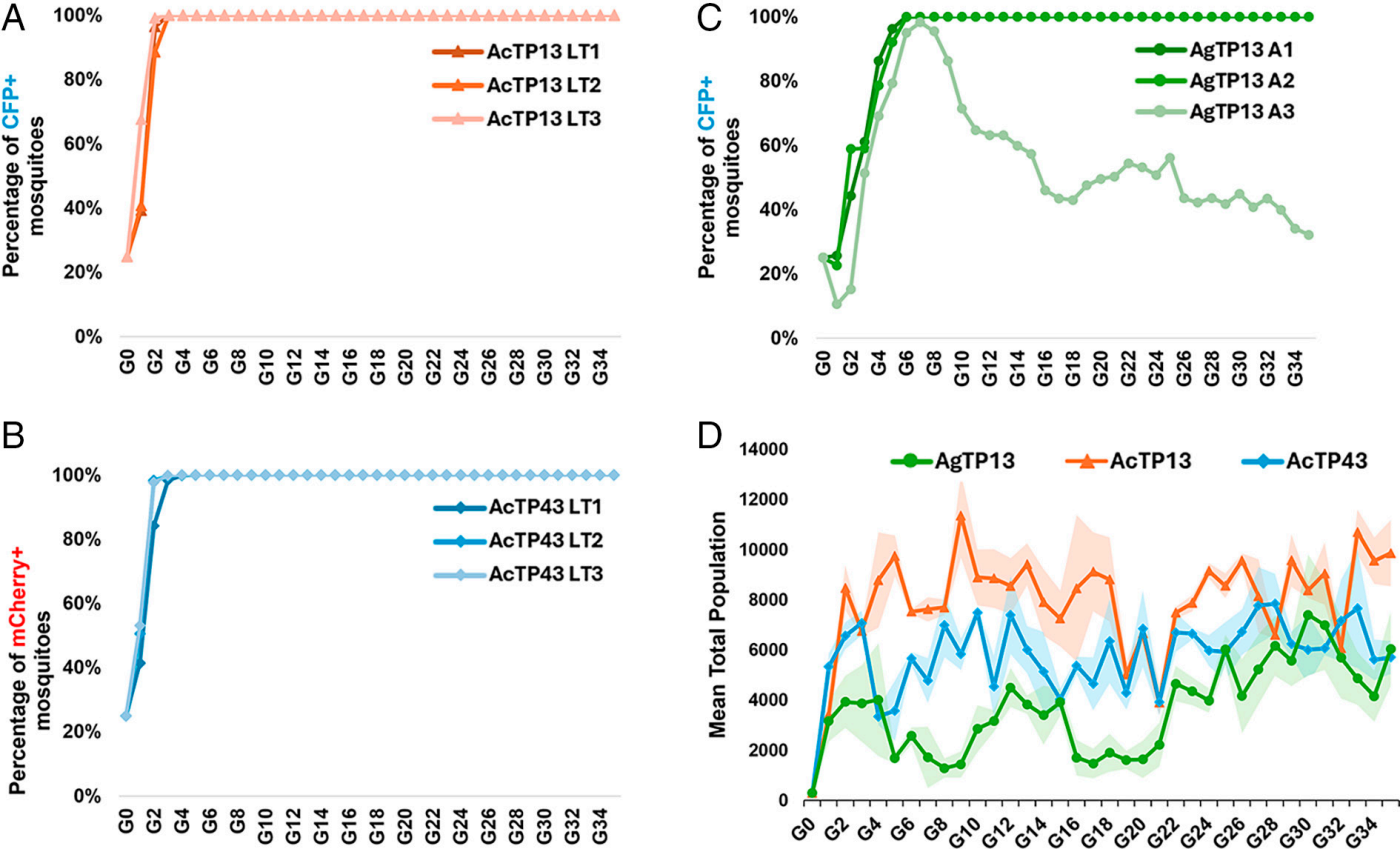
Drive dynamics and population sizes of long-term, non-overlapping generation cage trials. Three sets of triplicate cages were set up with 75 drive homozygous male (AcTP13, AcTP43, or AgTP13) along with 75 wild-type (WT) males and 150 WT females of (WT-Mopti for AcTP13 and AcTP43 cages; WT-X1 for AgTP13 cages) for 1:1 male release ratio. Totals of 600 to 800 mosquitoes were screened at each generation for the corresponding fluorescent marker of the drive system (CFP for AgTP13 and AcTP13; mCherry for AcTP43). (*A*) AcTP13 cages LT1, LT2, and LT3 drive dynamics (brown-shaded line, triangle markers). (*B*) AcTP43 cages LT1, LT2, and LT3 drive dynamics (blue-shaded line, diamond markers). (*C*) AgTP13 cages A1, A2, A3 drive dynamics (green-shaded line, circle markers). (*D*) Average total population sizes for each cage trial at every generation. Shaded areas are maximum and minimum counts among the triplicate cages. AgTP13 G0-13 data from Carballar-Lejarazú et al. (6) are used with permission.

Drive allele frequency in *A. coluzzii* AcTP13 cages (LT1-LT3) went from 25% in the release generation, G0, to 39.25% (250/637, LT1), 40.8% (274/672, LT2), and 67.59% (536/739, LT3) in the subsequent G1 generation (Fig. 1*A* and *SI Appendix*, Fig. S1*A* and Tables S1–S3). The drive systems then spread quickly with all three cages reaching 100% CFP^+^ at G3 (“full introduction”), indicating that all mosquitoes carry at least one copy of the drive (hemizygous). All mosquitoes in the screened samples reached phenotypic homozygosity at G4 indicated by red-eye phenotypes resulting from drive insertions into both copies of the *cardinal* gene target alleles (a red-eye phenotype also could result from a heteroallelic combination of the inserted drive and a nonfunctional, *cd*^−^, mutation, but subsequent molecular analyses found no evidence of this in these cages). Once fixed, drive homozygosity was maintained in all three AcTP13 cages until the end of the trial at 2 y (35 generations), with no drive-resistant or drive-negative animals recovered from any cage. A high rate of introduction and long-term stability also was observed in *A. coluzzii* AcTP43 cages (LT1-LT3), with first-generation, G1, mCherry^+^ percentages of 41.3% (296/716, LT1), 50.5% (307/608, LT2), and 53.0% (374/705, LT3), drive homozygosity achieved at G4-G5 and stability maintained throughout the trial (Fig. 1*B* and *SI Appendix*, Fig. S1*B* and Tables S4–S6). The *A. gambiae* AgTP13 cages showed lower initial drive allele frequencies compared to AcTP13. The early (G0-G13) AgTP13 cage results (A1-A3) were reported previously and the trials continued here through G35 (6). All three cages had low G1 CFP^+^ percentage of 25.7% (155/603, A1), 22.7% (131/578, A2), and 10.6% (65/616, A3) (Fig. 1*C* and *SI Appendix*, Fig. S1*C* and Tables S7–S9). Cages AgTP13-A1 and A2 reached 100% CFP^+^ at G6-G7, homozygosity at G7-G8, and remained so through G35. Cage A3 reached a maximum of ~98% CFP^+^ at G7 and drive allele percentages then fluctuated and declined as mutant, functional drive-resistant alleles accumulated.

Population sizes fluctuated among the cages and generations with AcTP13 cages having the largest with averages of 8,309 (LT1), 8,083 (LT2), and 8,029 (LT3) mosquitoes per generation (excluding cage founders) (Fig. 1*D* and *SI Appendix*, Tables S1–S3). AcTP43 cages had lower averages of 5,272 (LT1), 5,840 (LT2), and 6,660 (LT3) mosquitoes per generation (Fig. 1*D* and *SI Appendix*, Tables S4–S6). AgTP13 cage averages were the smallest with 3,681 (A1), 3,118 (A2) and 4,457 (A3) mosquitoes per generation (Fig. 1*D* and *SI Appendix*, Tables S7–S9). A total of 1,873,420 mosquitoes were scored from all nine cage trial replicates.

### Molecular Stability.

Drive system long-term molecular stability was determined using genomic DNA, total RNA, and amplification protocols (PCR, RT-PCR) with specific oligonucleotide primers (*SI Appendix*, Table S10). The results validate the proper insertion of the drive cassette into the mosquito genome, the effector sequences remaining free of mutations and the expression of the effectors at a postbloodfeeding time appropriate for parasite-blocking effects. Assessments of AcTP13 and AcTP43 mosquitoes were carried out at “Time zero,” the start of the cage trial, and at G18 (Year 1), and at G35 (Year 2). Time zero samples were collected from the siblings of the G0 homozygous drive males released into the cages. AgTP13 cages were assessed with G13, G24, and pooled G33-35 samples.

**Fig. 2.**
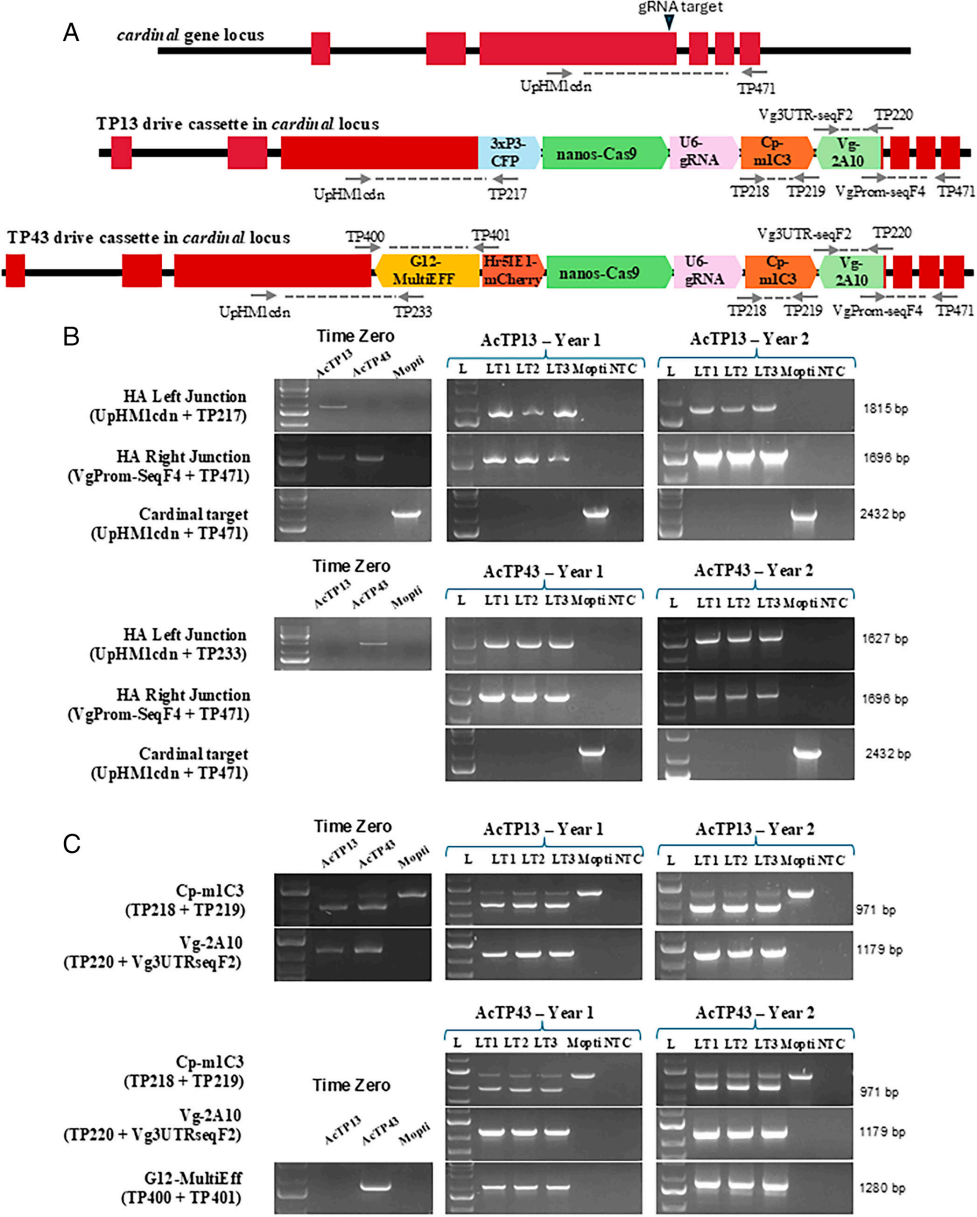
Schematic representation and drive stability validation of AcTP13 and AcTP43 cage trials. (*A*) Schematic representation of *cardinal (cd)* gene locus, TP13, and TP43 drive system. Components include *cd* gene exons (maroon blocks) with target site for gRNA; drive components *nanos*-Cas9 (green block) and U6-gRNA (pink block); antiparasite effectors Cp-m1C3 (orange block), Vg-m2A10 (light green block), G12-*MultiEff* (yellow block); and the eye marker 3xP3-CFP (blue block) and Hr5IE1-mCherry (red block). Gray arrows indicate primers for PCR and a gray dotted line indicates the amplified product. (*B*) Gene amplification validation of drive system in AcTP13 and AcTP43 cage trials at Time zero, Years 1 and 2 time points. Left and right junctions between the drive cassette and the *cd* homology arm were amplified to verify proper drive system insertion. Amplification of a region of the *Agcd* gene spanning the gRNA target site indicated the disruption of *cd* gene due to drive insertion. (*C*) Amplification of effectors cassette using primers between 5’ untranslated region (UTR) and 3’UTR for sequencing of the effectors m1C3, m2A10, and *MultiEff* at Time zero, Years 1 and 2 timepoints. L: molecular weight ladder. NTC: non-template control.

All AcTP13 and AcTP43 G18 and G35 cage samples were confirmed molecularly to be CFP^+^/*cd^−^* or mCherry^+^/*cd^−^*, respectively. Amplifications of the left and right junctions between the drive cassette and the *cardinal* locus produced amplicons in all samples with predicted sizes consistent with correct drive-system genomic integration (Fig. 2 *A* and *B*). A region of the *cardinal* gene spanning the gRNA target site also was amplified and all samples produced amplicons with predicted sizes consistent with disruptions caused by the integration of the drive system.

Analysis of AgTP13 CFP^+^/*cd*^−^ samples from cages A1 and A2 across generations also showed proper integration of the drive system at the target locus and absence of the intact *cardinal* gene due to drive insertion (*SI Appendix*, Fig. S2*A*). In contrast, the A3 cage population comprised mosquitoes with different phenotypes throughout the duration of the trial (*SI Appendix*, Fig. S1*C*). Analysis of CFP^+^ samples (CFP^+^/*cd*^+^, CFP^+^/*cd*^−^, and CFP^+^/tear) showed the expected presence of the drive insertion and its absence was confirmed in all CFP^−^ samples (CFP^−^/*cd*^+^ and CFP^−^/*cd*^−^) (*SI Appendix*, Fig. S2*A*). Previous molecular analyses of early-generation G8-G11) CFP^+^/*cd*^+^ individuals revealed genotypes comprising a drive allele paired with either a WT allele or an in-frame functional mutant allele, but all WT alleles were converted via homology-directed repair (HDR) at later generations and all *cd*^+^ phenotypes resulted from functional mutant alleles (frequencies and sequences shown in *SI Appendix*, Tables S11 and S12, respectively) (6). While two functional mutations were identified at generations prior to G13, only a small insertion, three base-pairs (bp) in length, persisted while a larger 24bp insertion was not recovered later. An out-of-frame, nonfunctional mutation was detected in CFP^−^ samples (CFP^−^/*cd*^+^ and CFP^−^/*cd*^−^) opposite one of the functional mutant alleles.

Effector sequences were monitored to detect any potential molecular lesions that would disrupt parasite-blocking capacity. The TP13 drive cassette contains two engineered genes encoding anti-*P. falciparum* effector single-chain antibodies (scFvs), m1C3 targeting ookinetes and m2A10 targeting sporozoites, that are regulated respectively by the *A. gambiae Carboxypeptidase A* (*AgCPA*, [*Cp*]) and *A. stephensi Vitellogenin 1* (*AsVg1*, [*Vg*]) gene promoters and 5- and 3-end untranslated regions (UTRs) (Fig. 2*A*) (6, 14, 15). In addition to these scFvs, the TP43 drive system also has a multiple effector cassette (*MultiEff*) with the coding sequences of four antimicrobial peptides (AMPs), melittin, shiva1, transportan 10 [TP10] and scorpine, and the enolase-plasminogen-interaction-peptide (EPIP) under the control of the *G12* gene promoter (7, 16). TP10 and EPIP are present as tandem copies of two and four DNA coding sequences, respectively. Amplification of DNA samples from Time zero, Years 1 and 2 mosquitoes with primers spanning the 5’- and 3’-end UTRs of the *Cp*, *Vg*, and *G12* promoter elements produced amplicons with expected sizes consistent with the whole m1C3, m2A10, and *MultiEff* gene coding sequences, respectively (Fig. 2 *A* and *C* and *SI Appendix*, Fig. S2*B*). The endogenous *Cp* gene also was amplified along with the m1C3 transgenes due to primer design. No structural mutations were detected in m1C3 and m2A10 in all AgTP13, AcTP13, and AcTP43 samples. Similarly, no structural mutations were recovered in the *MultiEff* constructs in AcTP43 cages LT1 and LT2 samples but a mutation resulting in the loss of one of the TP10 monomers was recovered from cage LT3 as early as G4 (8%, 4/50) and accumulated to 35% (36/104) by G35 (*SI Appendix*, Fig. S3).

**Fig. 3.**
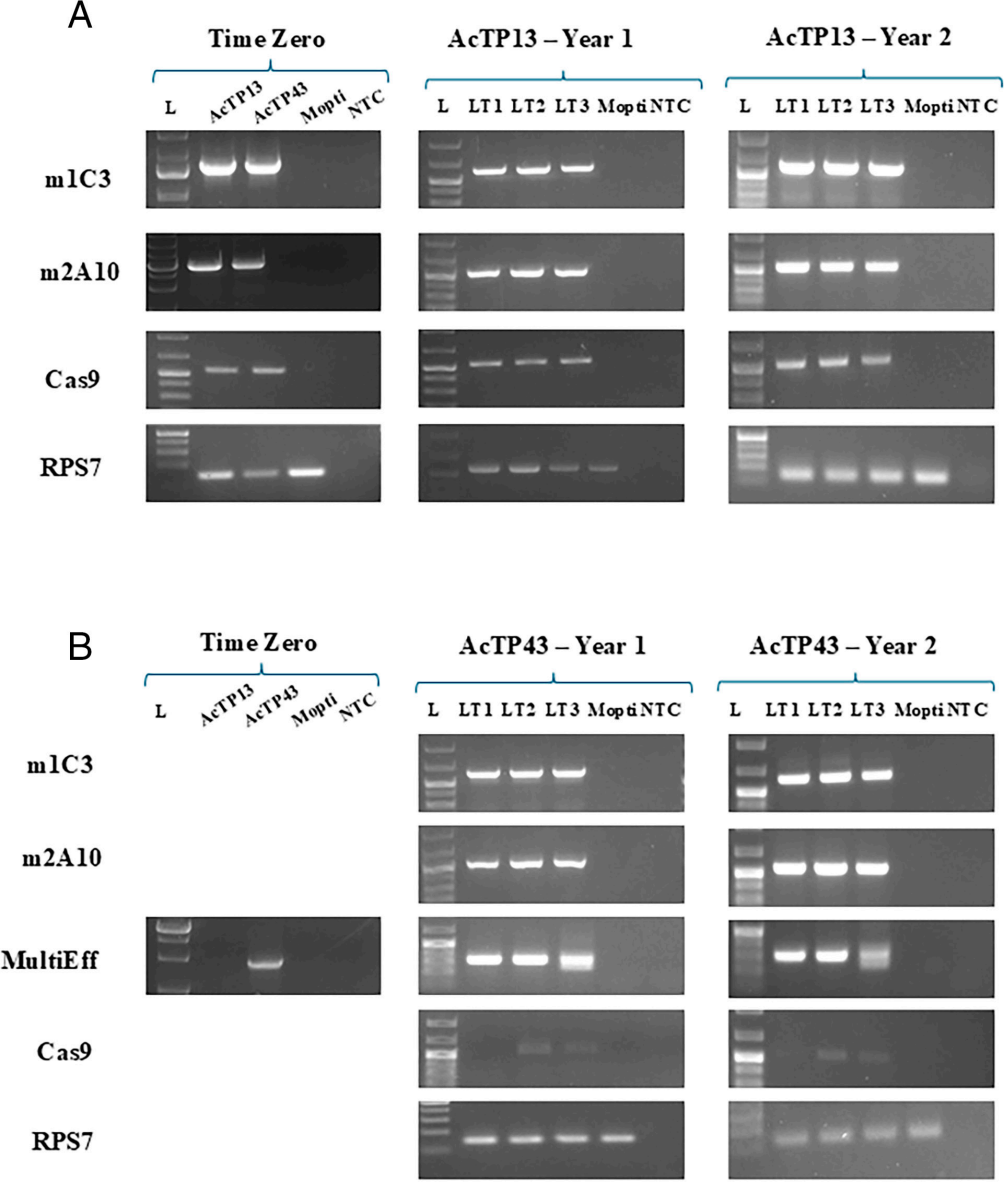
Expression analyses of antiparasite effectors and Cas9 in AcTP13 and AcTP43 cage trials. Gene- specific RT-PCR of m1C3, m2A10, *MultiEff*, and Cas9 driven by *Cp, Vg*, *G12*, and *nos* promoters, respectively, in AcTP13 (*A*) and AcTP43 (*B*) cage trials. Twenty female mosquitoes were collected from each cage at 24 h post blood meal and extracted for total RNA for RT-PCR templates. Ribosomal protein RPS7 was used as a control. The diffuse moieties in AcTP43-LT3 cage samples are due to the TP10 variants (described in *SI Appendix*, Fig. S3). L: molecular weight ladder.

The expression of m1C3, m2A10, *MultiEff*, and Cas9 mRNAs is driven in different tissues by blood-meal inducible promoters with different expression profiles. RT-PCR analyses of total RNA extracted from pooled samples (20 each) at Time zero, Years 1 and 2 whole adult females collected at 24 h post blood meal showed stable accumulation of m1C3 and m2A10 transcripts in all AcTP13, AcTP43, and AgTP13 samples (Fig. 3 and *SI Appendix*, Fig. S4). *MultiEff* transcript abundance also was consistent in AcTP43 samples despite the LT3 cage TP10 monomer mutation that produced a diffuse band at later time points due to the alternative size variants. Cas9 transcription driven by the *nanos* gene promoter and regulatory sequences was stable in AcTP13 samples but the AcTP43 mosquitoes showed qualitatively lower Cas9 accumulation at Year 2 evidenced by less dense staining of the amplicons in the gel and this is being followed up in related work. No Cas9 RT-PCR data are available for AgTP13 because this experiment was not planned at the time those lines were developed (6).

### Off-Target-Effects.

Previous assessments of off-target effects of the core AgNosCd-1 gene-drive system identified five potential sites using an algorithm that searched the whole genome for DNA sequences with nucleotide identities similar to the chosen target site (8). However, only the “off-target 1” variant on the second chromosome exhibited any cleavage in an in vitro assay and a low frequency, ~3 × 10^−3^ % (4/149,369 reads) of possible Cas9-induced small deletions at the predicted cut site. Off-target 1 site integrity was examined here as the most-likely “sentinel” site for monitoring any potential effects over the duration of the experiments. DNA from 20 mosquitoes from each trial cage at Time zero and Years 1 and 2 were pooled as one group. Gene amplification and next-generation sequencing (NGS) showed that the combined average percentage of indels in the DNA sequences, 1.74 × 10^−1^% (8,842,649 reads) was similar to the comparable WT controls, 1.77 × 10^−1^% (1,650,839 reads), supporting the conclusion that there was no activity of the Cas9–gRNA complex on this sentinel locus during the cage trials (*SI Appendix*, Table S13).

A single white-eyed/mCherry^+^ male was recovered at Time zero in female lineage progeny from AcTP43 drive efficiency experiments followed by eight males at Year 1 and none at Year 2 (*SI Appendix*, Table S14). Outcrosses and subsequent backcrosses provided evidence that the white-eye phenotype resulted from a recessive X-linked mutation (*SI Appendix, Text, Off-Target Effects*). Molecular analyses revealed an X chromosome deletion ~7.1 kg-base pairs (kb) in length that encompassed completely the *scarlet* gene (ACMO_005291) (*SI Appendix*, Fig. S5). The DNA surrounding the *scarlet* locus has three repeat domains (RD1-3), each 587bp in length, flanked by 13 bp inverted repeat sequences (CCCAAGTGCCACA**/**TGTGGCACTTGGG; *SI Appendix*, Table S16). The junctions of the deleted DNA are localized to RD2 and RD3 with single nucleotide polymorphisms characteristic of each in the sequence spanning the deletion (*SI Appendix*, Fig. S5 and Table S16). BLAST analysis of the repeat DNA showed high sequence identity (96 to 97%) with *A. coluzzii* genes encoding a beta-alanine-activating enzyme (LOC120955186) and the BTB/POZ domain-containing protein 9-like (LOC120952619), neither of which appear to have a direct role in the eye pigment synthesis pathway involving the *scarlet* gene product (17–19). The data support the conclusion that this deletion is a spontaneous mutation not related to the activity of the gene-drive system.

### Initial and Long-Term Drive System Efficiency.

Functional drive efficiency experiments were performed at Time zero (AcTP13 and AcTP43), and Years 1 and 2 (AgTP13, AcTP13 and AcTP43) to assess the long-term ability of the drive system to move itself and associated cargo into target alleles. Drive efficiency is determined by gene-drive inheritance, GDI, measured as the percentage of CFP^+^ or mCherry^+^ progeny of the total in any cross where one or both parents carry one or two copies of the gene-drive system, and the percentage of progeny inheriting an HDR-mediated “converted” gene drive target allele, cGDI, calculated as the percentage of progeny carrying the gene-drive system in excess of what would be expected by Mendelian segregation of the drive from a wild-type allele in mosquitoes hemizygous for the gene-drive system outcrossed to wild-type mosquitoes (6, 9).

Time zero assessments were performed with the progeny of siblings of the released gene-drive males and provide the baselines for comparisons with datasets from the following years. Homozygous male siblings of the *A. coluzzii* released males were first outcrossed to WT-Mopti females to generate hemizygous males and females to be used as parents (parental generation) in the drive efficiency experiments and all subsequent crosses in the experiments were performed with hemizygous offspring with the appropriate phenotype (wild-type eye, presence of drive system marker) as described (6, 8).

AcTP13 and AcTP43 had high drive inheritance and conversion at Time zero in both male and female lineages as indicated by >99% of F1 offspring inheriting a copy of the drive from either male or female hemizygous drive-carrying parents. (Table 1 and *SI Appendix*, Tables S17 and S18). A slight reduction in drive efficiency in F1 daughters from female lineages was observed in both AcTP13 and AcTP43 strains where only 96.9% and 95.5% of the F2 offspring, respectively, inherited a copy of the drive. However, drive efficiency remained high among all other F2 progeny (drive inheritance range = 97.8 to 99.9%). Both paternal and maternal effect-generated phenotypes were present in both gene-drive lines and lineages with low frequencies (<1% offspring) of *cd*^−^/red-eye and mosaic tear-eye individuals (range = 4.3 to 13.1%) in the female lineages, while the male lineages had only a small frequency of tear-eye individuals (<1.5%). Proportions of progeny with parental effect-generated phenotypes were similar to those observed previously in the original analyses of both lines (6, 7).

**Table 1.** Gene drive efficiencies in long-term cage trial mosquitoes

Male-founder lineage efficiencies^*^						
Cage trial	Time zero		Year 1		Year 2	
	GDI%	cGDI%	GDI%	cGDI%	GDI%	cGDI%
AcTP13	99.3–99.9	99.2–99.9	98.2–100	96.3–100	99.8–100	99.5–100
AcTP43	99.5–100	99.0–100	99.8–100	99.6–100	97.8–100	95.5–100
AgTP13	ND		98.3–100	96.5–100	99.8–100	99.6–100
Female-founder lineage drive efficiencies^*^						
Cage trial	Time zero		Year 1		Year 2	
	GDI%	cGDI%	GDI%	cGDI%	GDI%	cGDI%
AcTP13	96.9–99.2	93.9–98.4	98.0–99.8	95.4–99.6	97.9–99.3	95.8–98.6
AcTP43	95.5–99.7	90.9–99.2	97.7–99.9	95.4–99.8	98.8–99.5	88.2–99.5
AgTP13	ND		92.5–98.4	85.0–96.8	93.5–99.0	86.9–98.0

^*^Gene-drive efficiencies are assessed by calculating the percentage of total progeny inheriting the gene-drive allele (GDI) and the percentage of progeny carrying the gene-drive system in excess of what would be expected by Mendelian segregation of the parental drive and wild-type alleles (cGDI) (9). Male lineage include male founder F0, male progeny F1 from male founder, and male progeny F1 from female founder lineages. Female lineage include female founder F0, female progeny F1 from male founder, and female progeny F1 from female founder lineages. Ranges were combined from two generations of outcrosses. ND, not done here.

Samples of AcTP13 and AcTP43 homozygous males were collected from the cages at Years 1 and 2 and outcrossed to WT-Mopti females to generate hemizygous males and females for drive efficiency experiments. AgTP13 homozygous males also were taken at the Year 1 and 2 time points and mated to WT-X1 females. Drive efficiencies at Years 1 and 2 remained high for both male and female AcTP13 and AcTP43 lineages and were comparable to the results obtained for Time zero (Table 1). Drive inheritance of the parental drive hemizygous males and females was high with >99% of the progeny inheriting a copy of the drive (*SI Appendix*, Tables S19–S22). The slight decrease in drive efficiency in F1 daughters from female lineages was still present at Year 1 and 2 although less pronounced than at Time zero with ~98% of their progeny inheriting a copy of the drive compared to ~96% at Time zero. Both male and female lineages had evidence of paternal and maternal effect-generated phenotypes although the proportions of these phenotypes at Year 1 and 2 did not differ from those observed at Time zero. The *cardinal* red-eye phenotype remained rare (<1%) in female lineages and the tear-eye phenotype was present in variable proportions (2.8 to 10.6%), similar to what was observed at Time zero. The tear-eye phenotype in the male lineage progeny was observed in approximately the same proportion, <1.6%, as Time zero.

Previous work had shown high AgTP13 drive efficiency with male lineages having inheritance rates of 99.5 to 100% and female lineages with 85.5 to 96.9% (6). Similar to AcTP13 and AcTP43, there was high drive inheritance in the Year 1 parental drive hemizygous males and females, 100% and >98.4%, respectively (Table 1 and *SI Appendix*, Tables S23 and S24). As observed with AcTP13 and AcTP43, the F1 daughters of female lineages had a reduction in drive inheritance with ~93% of their offspring inheriting a copy of the drive at Years 1 and 2. This is lower than what was observed for AcTP13 and AcTP43 and the difference may result from the genetic background. Another difference in the AgTP13 drive efficiency compared to AcTP13 and AcTP43 is a higher rate of paternal- and maternal-effect phenotypes in the female lineages. The *cardinal* red-eye phenotypes generated in the AgTP13 female lineages were present in 0.35 to 1.3% of the progeny while tear-eye phenotypes were present in 8.8 to 24.5%, a value nearly double that of the AcTP13 and AcTP43 lines. While these phenotypic proportions were higher than those for the same time points in AcTP13 and AcTP43, there were no marked changes of these phenotypes from Year 1 to Year 2 in the AgTP13 cage experiments.

### Parasite Suppression.

A critical requirement for field applications is that the parasite-blocking efficacy persists in mosquitoes at levels that still impacts transmission (6, 7). Parasite challenge assays were conducted by feeding infectious, *P. falciparum* (NF54 strain) gametocyte-containing blood meals to samples of homozygous AcTP13, AcTP43, AgTP13 females from the cages at Years 1 and 2 and WT-Mopti and WT-X1 controls. The protocol was adapted from previous work and provides two additional, noninfectious blood meals at intervals after the initial infection (6, 7). Midguts from bloodfed females were dissected at 7 to 8 d postinfection (dpi) to determine oocyst infection prevalence and median and mean intensities of infection, and salivary glands of another cohort from the same feeding were dissected 14 to 16 dpi to obtain the same information for sporozoites.

**Fig. 4.**
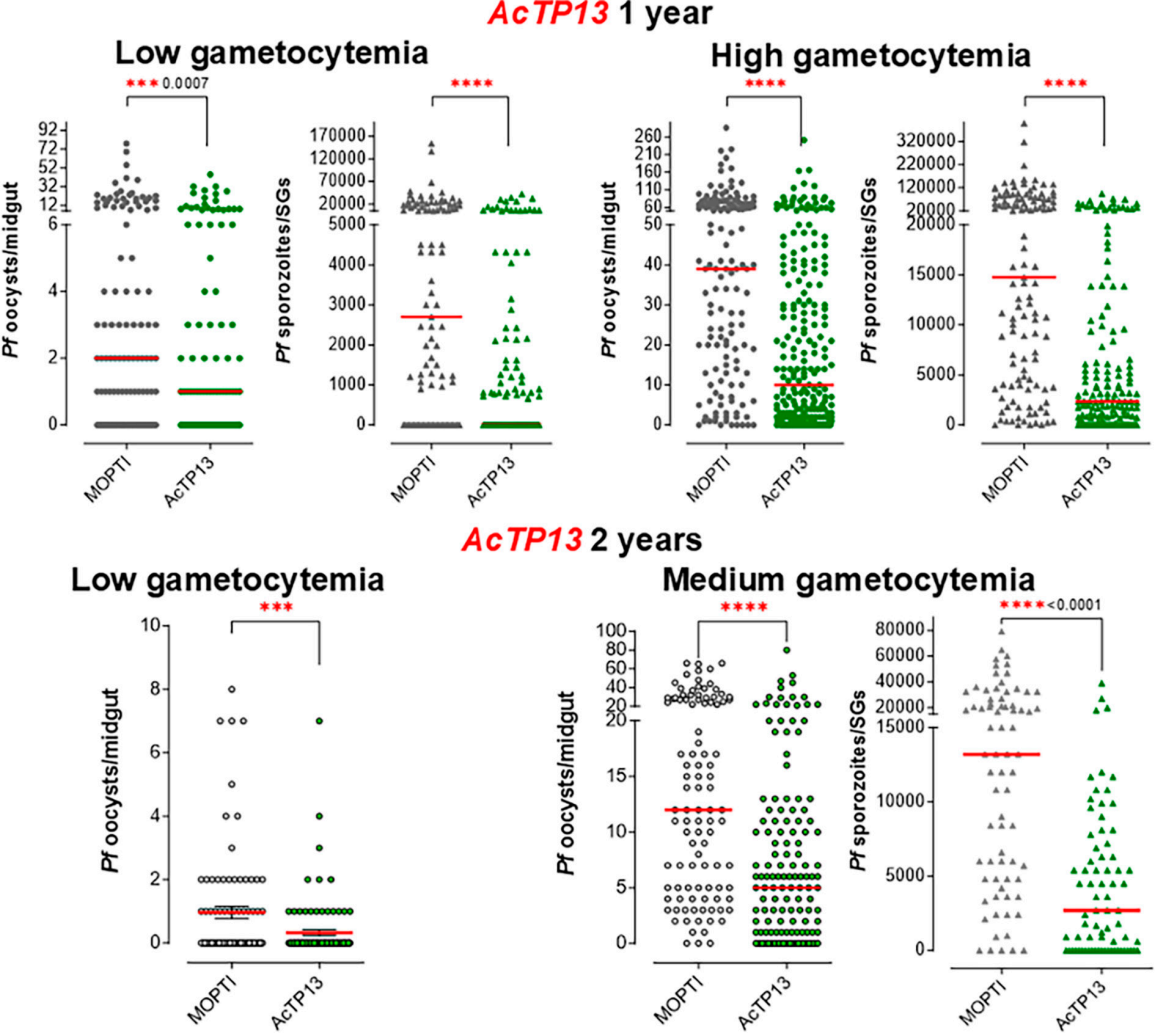
Impact of AcTP13 effector molecules on oocysts and sporozoites infection intensities after 1 and 2 y in colony culture. AcTP13 and MOPTI (wild-type control) adult females were fed infectious blood meals with Low (0.01 to 0.05%), Medium (0.1 to 0.2%), and High gametocytemias (0.3%) after one (1) or two (2) years in long-term culture. Midguts (midgut) and salivary glands (SGs) were dissected to determine oocyst and sporozoite numbers, respectively, and AcTP13 samples compared with controls. Each dot represents the parasite load in an individual tissue sample and the horizontal bars in the *Top* panels indicate the medians. The horizontal bars in the bottom Low gametocytemia panels denote the mean ± SE, as the medians are zero for both groups and therefore cannot be meaningfully compared. Statistically significant differences were assessed using two-tailed Mann–Whitney *U* tests. Exact *P* values are shown in the figure, along with significance annotations (*P < 0.05; ***P < 0.001; ****P < 0.0001).

Homozygous AcTP13 mosquitoes challenged at Year 1 with low (0.01 to 0.05%) or high (0.3%) gametocytemias (percentage of gametes in infected blood preparations) showed significant reductions in prevalence and median intensity of infection (Fig. 4, Table 2, and Dataset S1). Oocyst and sporozoite infection prevalence numbers in both challenge regimens did not reach the benchmark ≥32% reduction predicted to fully impact parasite transmission (20), however, the reductions were still statistically significant (oocysts, 28.0%, *P* = 0.0022; sporozoites, 29.5%, *P* = 0.0032). Nine (9/112) of the Year 1 low gametocytemia and 35 of 161 of high gametocytemia samples had >10,000 sporozoites in their salivary glands, another benchmark threshold proposed to impact transmission (21, 22). Notably, the Year 2 low-challenge (0.05 to 0.1% gametocytemia) assay showed significant parasite reduction, with a 53.5% decrease in oocyst prevalence (*P* = 0.0018) and a 69.5% reduction in mean oocysts intensity (*P* = 0.0006). Corresponding sporozoite numbers also were significantly lower, with prevalence showing a 52.1% reduction (*P* = 0.0418), mean sporozoite loads showing an 80.3 % reduction (*P* = 0.0139), and no salivary gland samples having >10,000 sporozoites. Technical issues resulted in smaller sample sizes for Year 2 challenges, with 48 Mopti control and 53 AcTP13 mosquito analyzed, and this is reflected in the statistical outcomes. Although the gametocytemia used in this assay was much higher (~twofold to fivefold) than in Year 1, overall *Plasmodium* infectivity was lower (with a mean of 1.1 and a median of 0 oocysts in the Mopti controls). Sporozoite infection prevalence in Mopti controls also was relatively low at 35.4%, compared with ~70% in Year 1. Therefore, the absence of any AcTP13 mosquitoes with sporozoite loads >10,000 is likely attributable primarily to the reduced parasite infectivity rather than stronger parasite-blocking activity by the mosquitoes. A parallel medium-challenge assay, performed with blood meals containing 0.1 to 0.2% gametocytemia, resulted in highly significant reduced oocyst and sporozoite prevalence (*P* < 0.0001 and *P* = 0.0002, respectively). However, the absolute reduction in sporozoite infection prevalence was 26.1%, which is below the 32% reduction benchmark, consistent with a more limited epidemiological relevance despite its statistical significance. Nine of 74 samples had sporozoite counts >10,000 per gland pair. The combined data confirm that the AcTP13 effector molecules still have significant parasite-reduction capabilities after 2 y of cage maintenance.

**Table 2.** Summary of AcTP13 Years 1 and 2 parasite challenge assays

Year 1												
Gametocytemia	Low (0.01 to 0.05%)						High (0.3%)					
	Oocysts			Sporozoites			Oocysts			Sporozoites		
	Mopti	AcTP13	Red.	Mopti	AcTP13	Red.	Mopti	AcTP13	Red.	Mopti	AcTP13	Red.
n	108	114		99	112		142	233		119	161	
Prevalence	73.1	52.6	28.0%	69.7	49.1	29.5%	95.1	81.1	14.7%	96.6	75.2	22.2%
Fisher’s		0.0022			0.0032			<0.0001			<0.0001	
Mean	8.1	4.0	51.0%	11,743	3,214	72.6%	50.2	23.0	54.1%	44,397	8,928	79.9%
Median	2	1	50.0%	2,700	0	*–*	39	10	74.4%	14,742	2,340	84.1%
Mann–Whitney		0.0007			< 0.0001			<0.0001			<0.0001	
Range	0–78	0–45		0–154,440	0–42,000		0–286	0–251		0–398,250	0–94,380	
Year 2												
Gametocytemia	Low (0.01 to 0.05%)						Medium (0.1 to 0.2%)					
	Oocysts			Sporozoites			Oocysts			Sporozoites		
	Mopti	AcTP13	Red.	Mopti	AcTP13	Red.	Mopti	AcTP13	Red.	Mopti	AcTP13	Red.
n	86	98		48	53		102	139		74	74	
Prevalence	39.5	18.4	53.5%	35.4%	17.0%	52.1%	97.1	78.4	19.2%	93.2	68.9	26.1%
Fisher’s		0.0018			0.0418			<0.0001			0.0002	
Mean	1.1	0.3	69.5%	1141.9	224.7	80.3%	17.6	8.7	50.7%	18,259.3	4,761.9	73.9%
Median	0	0	–	0	0	–	12	5	58.3%	13,200	2,700	79.5%
Mann–Whitney		0.0006			0.0139			<0.0001			<0.0001	
Range	0–10	0–7		0–12,300	0–2,880		0–66	0–80		0–79,200	0–39,000	

Gametocytemia, % of red blood cells with gametocytes; n, number of mosquito samples assayed; Prevalence, percentage of samples infected; Fisher’s, Fisher’s exact test of significance of prevalence; Mean, average number of parasite in all mosquitoes; Median, median number of parasites in all mosquitoes; Mann–Whitney, two-tailed Mann–Whitney *U* test (*P* values compare distributions between groups); Range, range of parasites in all samples from low to high; Red., percent decrease in the gene-drive mosquitoes compared to the wild-type controls.

**Fig. 5.**
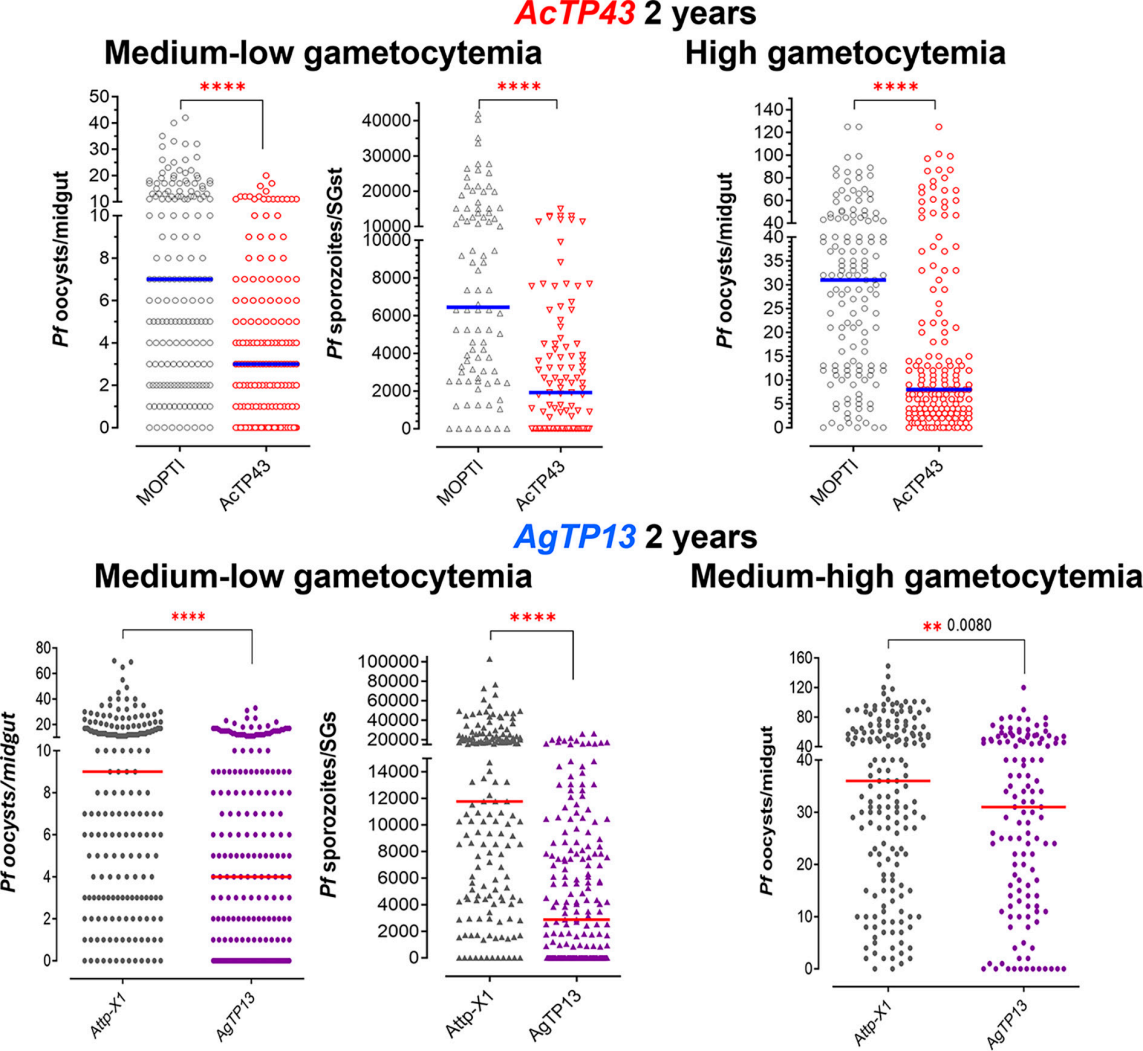
Impact of AcTP43 and AgTP13 effector molecules on oocysts and sporozoites infection intensities after 2 y in colony culture. AcTP43 and MOPTI (wild-type control) (*Top*) and AgTP13 and WT-X1 (wild-type control) adult females were fed infectious blood meals with Medium-low (0.05 to 0.1%), and High gametocytemias (0.3%) after 2 y in long-term culture. Midguts (midgut) and salivary glands (SGs) were dissected to determine oocyst and sporozoite numbers, respectively, and AcTP43 and AgTP13 samples compared with their respective controls. Each dot represents the parasite load in an individual tissue and the horizontal bars indicate the medians. Statistically significant differences were assessed using two-tailed Mann–Whitney *U* tests. Exact *P* values are shown in the figure, along with significance annotations (**P < 0.01; ****P < 0.0001).

Similar Year 1 *P. falciparum* challenge experiments were performed for AcTP43 and AgTP13 across six attempts. However, despite relatively high gametocytemia (0.3 to 0.5%), poor parasite infectivity produced low control infection prevalence and intensities, making the data insufficiently robust for statistical comparison; therefore, these data were not included. Results from Year 2 challenges of AcTP43 homozygous females with medium-low (0.05 to 0.1%) and high (0.3%) gametocytemias showed highly significant reduced oocyst prevalence in the medium-low regimen (24.8%, *P* < 0.0001) with the high regimen prevalence reduction, 5.3%, not significant (*P* = 0.0946) (Fig. 5, Table 3, and Dataset S1). The 59.1% and 57.1% reductions in the mean and median intensities of oocyst infections, respectively, in the medium-low gametocytemia regimen are statistically robust with the median being highly significant (*P* < 0.0001). The reduction in sporozoite prevalence in this regimen, 29.8%, is highly significant (*P* < 0.0001), as are the reductions in sporozoite mean (68.8%) and median intensities (70.2%, *P* < 0.0001). Furthermore, only 9 of 105 samples had salivary glands sporozoite numbers ≥10,000. As with AcTP13, these data show that the effector molecules in AcTP43 continue to show significant parasite-reduction capabilities after 2 y.

**Table 3.** Summary of AcTP43 and AgTP13 y 2 parasite challenge assays

AcTP43												
Gametocytemia	Medium-low (0.05–0.1%)						High (0.3%)					
	Oocysts			Sporozoites			Oocysts			Sporozoites		
	Mopti	AcTP43	Red.	Mopti	AcTP43	Red.	Mopti	AcTP13	Red.	Mopti	AcTP43	Red.
n	170	177		90	105		143	162		–	–	–
Prevalence	94.7%	71.2%	24.8%	92.2%	64.8%	29.8%	96.5%	91.4%	5.3%	–	–	–
Fisher’s		<0.0001			<0.0001			0.0946		–	–	–
Mean	9.1	3.7	59.1%	10,035.7	3,310.3	68.8%	33	18.7	43.5%	–	–	–
Median	7	3	57.1%	6,450	1,920	70.2%	31	8	74.2%	–	–	–
Mann–Whitney		<0.0001			<0.0001			<0.0001		–	–	–
Range	0–42	0–20		0–42,000	0–15,000		0–125	0–125		–	–	–

AgTP13												
Gametocytemia	Medium-low (0.05–0.1%)						High (0.3%)					
	Oocysts			Sporozoites			Oocysts			Sporozoites		
	WT-X1	AgTP13	Red.	WT-X1	AgTP13	Red.	WT-X1	AgTP13	Red.	WT-X1	AgTP13	Red.
n	210	234		167	194		174	127		–	–	–
Prevalence	93.8%	71.4%	23.8%	92.8%	63.9%	31.1%	98.9%	90.6%	8.4%	–	–	–
Fisher’s		<0.0001			<0.0001			0.0012		–	–	–
Mean	12.2	5.9	51.1%	17,016.8	5,077	70.2%	43.5	32.6	25.0%	–	–	–
Median	9	4	55.6%	11,760	2,880	75.5%	36	31	13.9%	–	–	–
Mann–Whitney		<0.0001			<0.0001			0.0080		–	–	–
Range	0–70	0–33		0–102,900	0–26,250		0–149	0–120		–	–	–

X1, WT-X1; Gametocytemia, % of red blood cells with gametocytes; n, number of mosquito samples assayed; Prevalence, percentage of samples infected; Fisher’s, Fisher’s exact test of significance of prevalence; Mean, average parasite number among all mosquitoes; Median, median number of parasites in all mosquitoes; Mann–Whitney, two-tailed Mann–Whitney *U* test (*P* values compare distributions between groups); Range, range of parasites in all samples from low to high; Red., percent decrease seen in the gene-drive mosquitoes compared to the wild-type controls. –: Sporozoite loads at high gametocytemia were not measured.

The results of medium-low (0.05 to 0.1%) and high (0.3%) gametocytemia parasite challenges of homozygous AgTP13 females are consistent with the severity of the challenge (Fig. 5, Table 3, and Dataset S1). Oocyst reductions in prevalence were greater in medium-low, 23.8% (*P* < 0.0001), than in the high, 8.4% (*P* = 0.0012) samples. Reductions in the oocyst mean (51.1%) and median (55.6%; *P* < 0.0001) intensities of infection in medium-low gametocytemia regimens also were higher than the corresponding values in high-regimen samples (mean, 25.0%; median, 13.9%; *P* = 0.0080). The reduction in sporozoite prevalence in the medium-low regimen, 31.1%, is highly significant (*P* < 0.0001), as are the reductions of sporozoite mean (70.2%) and median intensities (75.5%, *P* < 0.0001). However, 39 of 194 samples had salivary glands sporozoite numbers ≥10,000. While not as high as AcTP13 and AcTP43 strains, the effector molecules in the AgTP13 strain do provide significant reductions in parasite numbers after 2 y.

## Discussion

The long-term stability and efficacy of population modification gene drives for controlling parasite transmission are essential if they are to play a role in the malaria eradication agenda (23). However, despite rapid progress in drive engineering, well-designed population modification strains have only been available for the last few years, and as a consequence, there is a dearth of longitudinal studies that simultaneously track population-level dynamics, molecular cassette integrity, resistance allele emergence, and parasite suppression in multiple independent replicates and species backgrounds. We analyzed three strains of African malaria vector mosquitoes, AcTP13 and AcTP43 in *A. coluzzii* and AgTP13 in *A. gambiae*, in three replicates of nonoverlapping cage trials over a 2-y period equivalent to 35 generations. The trial design tests durability beyond that of short-term inheritance assays or single-cage studies, and more closely approximates the operational requirements of sustained performance after release used in modeling studies (4, 6). Stability was evaluated by monitoring maintenance of drive efficiency (gene drive inheritance and gene conversion, “homing”), population dynamics (size), molecular integrity of the gene-drive cassettes, generation and accumulation of mutant, drive-resistant target-site alleles, and effector gene antiparasite activity. This integrated evaluation framework represents a key advance by linking the genetic drive component to the public health-relevant endpoint, parasite suppression, over an extended timescale.

The results are encouraging and informative for future release protocol design and modeling. All lines were stable at 2 y for drive efficiency and parasite suppression with the exception of one AgTP13 replicate that accumulated a cleavage-resistant target-site mutation. All six *A. coluzzii* replicates achieved drive system fixation within three generations after release while the *A. gambiae* replicates were slower at the outset with two of the three cages taking six and nine generations to achieve fixation. However, once established, these two were stable through the end of the experiments. The emergence of resistance in the single AgTP13 replicate while all other cages remained stable highlights the value of replication for estimating operational risk.

The failure of the third AgTP13 replicate to achieve fixation provides an opportunity to identify factors that influence successful outcomes. A major initial factor is the ability of gene-drive carrying males to compete in the presence of wild-type males for mating with females. We had shown previously that the aggregate homozygous AgTP13 male fitness evaluated as the “likelihood to contribute to the next generation,” 22.4%, was significantly lower than the 50% expected if they were as competitive as their wild-type counterparts (hemizygotes contributed 48.8%) (6). In contrast, homozygous AcTP13 males were as likely to contribute as their wild-type competitors, 51.2%, and remarkably, hemizygotes were more likely to contribute, 64.1%. Hemi- (77.1%) and homozygous (56.2%) AgTP43 males also were more likely to contribute to the next generation in the presence of direct competition (7). These differences are evident in the cage trial data reported here in the percentages of marker gene phenotypes seen in the early generations of the cage trials. The slower initial AgTP13 drive dynamics appears to create conditions permissible to the formation of the nonfunctional and functional cleavage-resistant alleles seen in the third cage replicate.

The third AgTP13 cage replicate also provides evidence of competitive mating biases among mosquitoes carrying the drive-system and nonfunctional or functional mutant alleles. The predominant phenotypes at the end of this trial were CFP^+^ and CFP^-^ with wild-type (*cd*^+^) eye color in mosquitoes hemi- and homozygous for the 3 bp-insertion functional resistance allele described in the Results. The gradual increase of the CFP^−^/cd^+^ phenotype toward the end of the experiment is consistent with a load associated with the presence of the drive system allele with the decreased mating competitiveness of homozygous AgTP13 males providing a positive reproductive bias for the CFP^−^/*cd*^+^ individuals. Previous work found that homozygous AgTP13 females are not likely to bias inheritance of either allele types as they were found to be fitness-neutral when compared to wild-types (6). In the same study, hemizygous AgTP13 males and females, CFP^+^/*cd*^+^, also were fitness neutral when compared to wild-types. There also is the possibility of a secondary contribution of a positive fitness advantage of the functional resistant allele that became highly prevalent and this would further skew the population to higher proportions of CFP^−^ individuals. However, while the percentage of CFP^+^/*cd*^+^ mosquitoes is decreasing toward the end of the third cage replicate, they still represent a potentially impactful proportion of the population.

The genetic basis for the load that affects AgTP13 fitness is unclear. It could result from insertion site mutagenesis of the wild-type *cardinal* gene or expression effects of the gene drive system. Previous work with homozygous males of a strain, *Agcd^Δ11^*, carrying a nonfunctional 11 bp deletion in the target site, showed that they were as competitive as wild-type males for mating with females supporting the conclusion that the nonfunctional resistant allele does not confer a reproductive load, and that the homozygous *cd^−^*, red-eye phenotype would not be selected against in a mixed population (9). However, the loss of homozygous *cd^-^* mosquitoes in the third AgTP13 replicate stands in contrast to this finding and may reflect a pleiotropic effect of this deletion.

Some fitness effects could result from a load imposed by expression of drive-system components and this is consistent with the differences seen between the hemi- and homozygous AgTP13 and other males noted above. It is worth reiterating that these results are influenced by the genetic background because they are not as severe in the *A. coluzzii* strains. If this is an issue moving forward, it could be mitigated by incorporating DNA that results in a functional *cardinal* “rescue” allele as was done for the *cinnabar* gene target in *A. stephensi* or selecting a different target locus altogether (24).

Mutant drive-resistant target-site alleles that affected fixation were recovered only in the one AgTP13 replicate. A separate analysis of AgTP13 reported a relatively low average Cas9/gRNA-mediated germline mutation rate (0.21%) but noted that clustering effects can substantially amplify (twofold to sevenfold) mutant allele inheritance (9). The present data extend these findings by showing that this amplification can result in different cage outcomes despite the low average mutation rate, reinforcing the need to evaluate resistance risk in replicates rather than as single point estimates. The high-efficiency drive seen with the *A. coluzzii* genetic background strains may mitigate this effect by rapidly achieving fixation that results in the conversion of cleavable wild-type alleles into drive alleles that are no longer substrates for Cas9/gRNA cleavage (6). This hypothesis is supported by results of cage trials using different, 1:1 vs. 1:3, release ratios of gene-drive to wild-type males in which faster drive-system introgression generated no or few resistance alleles (6).

Drive dynamic efficiency assessed at the start and at years 1 and 2 remained stable, with high inheritance (92.5 to 100%) and conversion frequencies (85 to 100%) consistent with prior reports in anophelines (6, 7, 9, 25). Male-lineage inheritance and conversion generally exceeded female-lineage values, consistent with previously observed maternal effects that convert wild-type target alleles into drive-resistant alleles (6, 9, 12). However, the persistence of these sex-specific patterns without progressive loss over 35 generations supports long-term robustness against intrinsic germline constraints often invoked as failure modes (26).

Average population sizes differed but were consistent within each strain with *A. coluzzii* AcTP13 and AcTP43 maintaining larger populations than *A. gambiae* AgTP13. Previously reported life-table parameters, including development times, longevity and reproductive capacity, do not clearly explain these differences; therefore, they again likely reflect the baseline hardiness of the source strains rather than drive-specific effects (6, 7). The findings here highlight how long-term drive system performance can be shaped by background effects not readily predicted from single-trait measurements, and emphasize the need for extended population-level validation of candidate release strains.

With one exception, the molecular integrity of the drive systems was stable throughout the experiments. A loss of one of the two copies of the TP10 A-encoding DNA was observed in samples from one AcTP43 cage while the four tandem copies of EPIP remained stable throughout the duration of the experiments. Genes present in direct tandem copies are known to delete and insert additional copies as a result of chromatid misalignment and recombination during meiosis (27–29). The “genetic breakdown” of a conditional lethal strain in *Drosophila* was attributed in part to the presence of tandem duplications in the system (30). Thus, genetic constructs for manipulating insect phenotypes should avoid using tandem repetitive DNA sequences when possible. The deletion recorded here is a rare example of cargo restructuring in mosquitoes that was only detectable through longitudinal monitoring and emphasizes the need for long-term surveillance that includes the effector cassette.

No off-target effects attributable to the drive system were detected in the sentinel off-target 1 locus over the duration of the experiments and background indel levels measured by NGS were comparable between controls and drive lines. The single sentinel locus was used because previous work showed that the next four candidate loci identified following a full search of the genomic sequences were uncleavable by Cas9/gRNA complexes and thus functionally equivalent to resistance alleles (8). The current data emphasize drive-system target specificity in the genomic context tested. A preliminary hazard assessment of autonomous gene-drive systems identified other possible genetic off-target outcomes not limited to the target site variant examined here, most notably deletions, inversions, and translocations (31). Comprehensive, whole genome scanning for these and other potential effects may be required in risk assessment portfolios.

The deletion mutation recovered in the X-linked *scarlet* locus is unlikely to represent an off-target effect of the drive system. The recovered DNA sequence is consistent with recombination in a repeat-rich region (potentially involving transposon-associated repeats), highlighting natural genome plasticity in mosquitoes and the need to evaluate rare structural variants in alternative contexts rather than assuming drive-associated causality (32).

Important translational considerations include what efficacy thresholds are required to meaningfully suppress transmission and how long antiparasite effector genes must remain active. Previously proposed threshold values (e.g., ≥32% reduction in oocyst prevalence over three transmission cycles and salivary gland sporozoite loads <10,000) provide practical targets for evaluating effector performance (6, 7, 9, 20–22). Although these numbers were derived from meta-analyses and animal model studies and were not intended for assessing population modification gene-drive systems, they provide early-phase targets for evaluating effector molecule efficacy. The varied gametocytemias and infection outcomes among the assays presented here prevent direct comparisons of all experimental groups, but those with low (AcTP13) and medium-low (AcTP43) challenges were close to meeting or met threshold value levels at 2 y that still could be expected to impact malaria prevalence. Notably, observing persistent reductions under low-gametocytemia conditions is particularly informative because these conditions may better approximate many natural transmission settings where infectious individuals often carry low gametocyte densities (33, 34). As expected from previous work, those with higher gametocytemias did not meet these values, but many showed statistically significant reductions in prevalence and intensities of infection at the 2-y mark (6).

Modeling predicted that a persistent effect over two transmission seasons (~2 y) is needed for epidemiologically significant reductions in malaria incidence (4). A key contribution of the work here is direct empirical evidence that the significant reductions in parasite prevalence and intensities of infection observed in AcTP13, AcTP43, and AgTP13 following challenges with low (“natural”) gametocytemia levels meet the 2-y goal while the drive systems remain intact and functional. This linkage between durability (inheritance, cassette integrity, resistance) and biological impact (infection outcomes) over 2 y helps bridge a key translational gap between gene-drive mechanics and malaria control relevance.

We recognize the limitations of laboratory-based work using long-colonized mosquito strains with little genetic variation challenged with parasite strains with low genetic complexity in fixed environmental conditions. However, the data support the conclusion that population-modifying gene drives can sustain meaningful transmission-blocking activity across a realistic range of parasite challenge intensities, while clarifying conditions under which efficacy decreases under high infection intensity pressures. By integrating multi-year cage performance, replicate-level resistance outcomes, longitudinal cassette integrity, and sustained antiparasite phenotypes across African vector backgrounds, this work provides a comprehensive durability benchmark that can inform risk assessment and the design of these and other next-generation multi-effector, resistance-resilient drive systems.

## Materials and Methods

Details of all strains, reagents, and procedures are provided in the *SI Appendix, Materials and Methods*.

### Ethics Statement.

All studies were performed in compliance with the requirements of the Guide for the Care and Use of Laboratory Animals of the NIH, and the Institutional Animal Care and Use Committee at Johns Hopkins University approved the protocol (permit no: M006H300). Mice were used only as a blood source for mosquito rearing. Commercial anonymous human blood was used for *Plasmodium* gametocyte cultures and infection assays and informed consent was not required.

### Mosquito Strains and Lines.

Wild-type control lines, *A. gambiae* X1 and *A. coluzzii* Mopti, and transgenic lines, AgTP13, AcTP13, and AcTP43, are the sources of all mosquitoes used here (6, 7).

### Long-Term Cage Trials.

Three independent replicate experimental cages of each transgenic line were used based on previous work (6, 13).

### Molecular Analyses.

Gene amplification-based protocols were used to validate gene-drive cassette integration, effector molecule integrity, and antimalarial effector expression, detect off-target effects, and characterize the white-eye mutation and a selection of wild-type and mutant (“exceptional”) alleles (6, 8). Sanger sequencing and/or next-generation sequencing (NGS) was performed on representative samples as described (6, 8).

### Drive Efficiency.

Drive efficiency experiments were performed as described previously and outcomes expressed as percentages of gene-drive inheritance (GDI) and HDR-mediated gene conversion, (cGDI) in progeny following crosses (6, 8, 9, 13).

### Parasite Challenge Assays.

Adult homozygous AgTP13, AcTP13, and AcTP43 female mosquitoes were used in multiple replicate parasite membrane feeding assays along with their respective control lines, WT-X1 and WT-Mopti, based on previous protocols (6, 16). Raw data are listed in Dataset S1.

### Statistics.

Statistical analyses, *Χ*^2^, Fisher’s exact, and the two-tailed Mann–Whitney *U* test, were used where appropriate with *P*-values < 0.05 considered significant.

## Supplementary Material

Appendix 01 (PDF)

Dataset S01 (XLSX)

## Data Availability

Previously published data were used for this work (6). Other data are included in the article and/or supporting information.
