# Supplementary material for: Long-term stability and performance of Cas9/guide RNA-based gene drives in anopheline mosquitoes
Source: Proc Natl Acad Sci U S A. 2026 Jul 6;123(28):e2605739123. doi: 10.1073/pnas.2605739123 (PMC13367772; doi:10.1073/pnas.2605739123)
Supplement: Supplementary file 1 — Appendix 01 (PDF) [file pnas.2605739123.sapp.pdf]

PNAS

Supporting Information for

Long-term stability and performance of Cas9/guide RNA-based gene drives in anopheline mosquitoes

Rebeca Carballar-Lejarazú, Yuemei Dong, Thai Binh Pham, Taylor Tushar, Drusilla Stillinger, Devin Ngoc Nguyen, Lorena Winokur, Mihra Tavadia, Mabel Tao, George Dimopoulos, Anthony A. James

Corresponding author: Anthony A. James
Email: aajames@uci.edu

This PDF file includes:

Supporting Information Text
Figures S1 to S5
Tables S1 to S24
Legend for Dataset S1
SI References

Other supporting materials for this manuscript include the following:

Dataset S1

Supporting Information Text

OFF TARGET EFFECTS

Discovery and analysis of a white-eye mutation

Mosquitoes with a white-eyed/mCherry$^+$ phenotype were recovered from the female lineage progeny of the AcTP43 drive efficacy experiments (SI Appendix Table S14). Four of the eight males recovered during Year 1 survived and were mated to WT-Mopti females (SI Appendix Table S15). In one experiment, one white-eyed male homozygous for the gene-drive system was outcrossed to 20 WT-Mopti females. All progeny were positive for fluorescent eyes (mCherry$^+$) and had wild-type colored eyes supporting the conclusion that the mutant allele responsible for the white-eye phenotype is recessive. A second outcross of the gene-drive hemizygous, wild-type eye color F1 female progeny with WT-Mopti males generated F2 progeny with high, 99.1% (563/568), gene-drive inheritance and high, 98.2% (279/284) target allele gene conversion. Only white-eyed males were recovered supporting the conclusion that the gene responsible for the phenotype is linked to the X chromosome and the lack of white-eye females is consistent with a recessive mutant allele. The small number of mosaic phenotypes recovered is inconsistent with previous observations of high frequencies of maternal effects in outcrosses of female gene-drive lineages[1-5]. The F2 progeny sex ratio (333♂/235♀, X^2 = 16.908, d.f.=1, p<0.0001) was biased significantly toward the recovery of males. An intercross of F2 mCherry$^+$/white-eye males with mCherry$^+$ wild-type eye females further confirmed the recessive property of the mutant allele as some F3 white-eye females were recovered, presumably as a result of heterozygotes present in the female F2 progeny. Here again, a significant bias in the sex ratio was seen (194♂/329♀, X^2 = 34.847, d.f.=1, p<0.0001), but this time favoring females. Gene amplification-based molecular analyses of white-eyed individuals with primer pairs TP678 and TP698 revealed an X chromosome deletion and the loss of the *scarlet* gene (ACMO_005291) (SI Appendix Figure S5 and Table S10).

MATERIALS AND METHODS

Mosquito Lines and Maintenance

The AgTP13, AcTP13 and AcTP43 mosquito gene drive lines used in these experiments have been described previously[1,2]. The wild-type control lines in which the gene-drive lines were generated are the *An. gambiae* X1 (WT-X and *An. coluzzii* Mopti strain (WT-Mopti, BEI Resources, MRA-763)[6]. Mosquitoes were maintained at 27 °C with 77% humidity and a 12-h day/night, 30-min dusk/dawn lighting cycle. Larvae were fed powdered fish food (TetraMin) mixed with yeast. Adults were provided *ad libitum* with water and a 10% (wt/vol) sucrose solution. Defibrinated rabbit blood (Colorado Serum) was provided using an artificial feeding apparatus (Hemotek) for blood meals.

Long-term cage trials

Three sets of triplicate experimental cages, each with a volume of 5,000 cm^3, were established with a 1:1 initial release ratio of 75 homozygous drive males (AgTP13, AcTP13, or AcTP43) to 75 wild-type (WT) males. In all cases, 150 WT females were subsequently introduced to achieve a balanced male-to-female ratio with a total population of 300 adults per cage. Subsequent generations were screened and analyzed following previously-described methods[1,7]. A total of, 300 randomly-selected second-instar (L2) larvae from each cage were removed every generation to a new cage for the subsequent generations, and an additional 800 randomly-selected L2 larvae were reared separately to pupae for sexing and phenotypic screening. Drive dynamics were assessed by screening AcTP13 and AgTP13 pupae for CFP marker gene fluorescence to determine the presence or absence of the drive system, while AcTP43 pupae were screened for the mCherry marker. Pupae also were screened for eye-color phenotypes (black, wild-type [WT, *cd*$^+$]), pale red-eye (*cd*$^-$) or mosaic (tear) for the intact *cardinal* gene or disruptions resulting from the insertion of the drive-drive system into the genome. The remaining larvae were scored for the total population count.

Molecular analysis

Molecular validation of gene-drive cassette integration and effector molecules integrity. Genomic integrations were verified by gene amplification analyses (PCR) using genomic DNA of cage-trial mosquito samples at selected generations as templates and gene-specific oligonucleotide primers (SI Appendix Table S10). Genomic DNA extraction, PCR reactions and sequencing procedures were carried as described[1]. The right-hand junction between the drive cargo of TP13

and TP43 and the *cardinal* gene left homology arm was verified by the primer pair VgProm-seqF4/TP179. Primers upHm1cdn/ TP179 were used to amplify a fragment of the *cardinal* gene spanning the insertion location of the drive cassette between the left and right homology arms to verify the presence of a WT allele in the hemizygous (one copy of the dominant CFP fluorescence gene) mosquitoes. The primer pair upHM1cdn/TP217 was used for the left-hand junction between the right homology arm and the TP13 cargo, while the left junction of TP43 cargo was amplified with the primer pair upHM1cdn/TP233. To validate the integrity of the effector molecules, primer pairs TP218/TP219, Vg3UTR-seqF2/TP220, and TP400/TP401 were used to amplify fragments from the 5'-end untranslated region (UTR) to the 3'-end UTR of m1C3, m2A10, and the *MultiEff* effector cassettes, respectively. None of the amplicons were sequenced because the drive systems remained functional.

RT-PCR for the antimalarial effector expression validation- Females from each cage trial at selected generations were bloodfed and collected after 24 hours for RT-PCR analysis of antimalarial effector and Cas9 gene transcription products. Total RNA extractions, cDNA synthesis and PCR reactions were carried out on pools of 20 mosquitoes from each group as described[1]. Synthesized cDNA was amplified with gene-specific primer pairs, TP236/TP237, TP240/TP241, RT-*MultiEff*-F/RT-*MultiEff*-R2, CO30/TP456 and RPS7-F/RPS7-R, to determine the expression of m1C3, m2A10, *MultiEff*, Cas9 and the ribosomal protein, RPS7, respectively.

Off-target amplifications **-** Amplicon EZ deep sequencing was used for off-target analysis of cage trial samples. Twenty mosquitoes from each trial cage at pre-determined time points (Time zero, Years 1 and 2) were pooled and sequenced as one group. WT-X1 and WT-Mopti were also included as control. Primers off-1-EZ-F and off-1-EZ-R were used to amplify the regions around the off-target 1 site on chromosome 2L for deep sequencing as described[5].

Scarlet gene amplifications

Genomic DNA from white eyed, mCherry$^+$ mosquitoes recovered from AcTP43 drive efficacy experiment at Year1 were extracted individually as described[1]. Genomic DNA samples from Mopti and AcTP43 *cardinal* red-eyed, mCherry$^+$ mosquitoes were used as controls. Amplification of the *scarlet* gene ACMO_005291 were performed using DreamTag PCR mix (ThermoFisher, USA) following the manufacturer's protocol with the primer pairs TP539/TP540 and TP541/TP542 for a 561 base pairs (bp) and 1,874 bp fragments, respectively (SI Appendix Table S10). Primer pairs, TP678/TP698 are spaced ~9.1kb apart on the X chromosomes in the wild-type undeleted genomic sequence and produced an ~2.0kb fragment using the *scarlet* deletion mutant DNA as a template. PCR products were resolved using 1% agarose gels.

Drive efficacy

Drive efficacy experiments were performed as described previously[1,4,5]. Briefly, 50 drive homozygous males collected from each replicate of each long-term cage experiment were outcrossed to their respective wild-type lines, WT-X1 for AgTP13, and WT-Mopti for AcTP13 and AcTP43, in order to create hemizygous drive-carrying males and females. Fifty of these newly-generated drive hemizygous males and females then were collected and outcrossed to members of the opposite sex from their respective wild-type line to establish male and female lineages. The total progeny from each mating were screened for eye color and the presence/absence of the respective drive-system fluorescent marker. Another wild-type outcross was performed on 50 randomly-selected offspring from each cross that appeared hemizygous for the drive-system based on their phenotype, (wild-type [cd^+]/CFP$^+$ for AcTP13 and AgTP13 or cd^+/mCherry$^+$ for AcTP43) to evaluate changes in drive inheritance or drive efficacy over multiple generations (Time zero, G18 [Year 1]) and G35 [Year 2]).

Parasite challenge assays and statistical analyses

Adult homozygous AgTP13, AcTP13 and AcTP43 female mosquitoes were used in parasite membrane feeding assays along with their respective control lines, WT-X1 and WT-Mopti. All mosquitoes were maintained under standard insectary conditions as described[6]. Briefly, mosquitoes were kept at 27 ± 1 °C, 70–80% relative humidity, with a 12:12 h light:dark photoperiod. Females were provided a 10% sucrose solution *ad libitum* until just prior to the infectious bloodmeal. The *P. falciparum* NF54 strain was maintained in human O$^+$ erythrocytes until mature stage V gametocytes were obtained approximately day 15. Gametocyte cultures were mixed with fresh human erythrocytes and serum to generate the desired challenge levels. Because mosquito infection outcomes are influenced by both gametocytemia and intrinsic gametocyte infectivity, challenge categories were defined by the resulting infection intensity in wild-type control mosquitoes rather than by gametocytemia alone. In general, infections with median control oocyst loads of <5, <10, <15, and >25-30 were classified as 'low', 'medium-low', 'medium', and 'high' challenge, respectively. Corresponding

gametocytemia ranges were empirically adjusted across assays and included 0.01-0.05% gametocytemia (representative of many natural transmission levels) for AcTP13 Year 1 low challenge, 0.05–0.1% for AcTP13 Year 2 low challenge and for medium-low challenges, 0.1-0.2% for medium challenges, and 0.3% for high challenges. Bloodmeals were maintained at 37 °C and offered to mosquitoes for 60 min using a membrane-feeding apparatus fitted with parafilm following previously established protocols[8]. Fully engorged females were separated within 1 h post-feeding and maintained on 10% sucrose for the duration of the parasite development period. Additional uninfected blood meals were provided at 5- and 10-d post infection to boost the blood meal inducible effector gene expression.

Oocyst counts were obtained from midguts dissected at 7-8 days post-infection, stained with 0.1% mercurochrome, and examined under a light microscope at 100x or 200x magnification. The number of oocysts per midgut was recorded for each mosquito. Sporozoite counts were obtained from salivary glands dissected at 14-16 days post-infection. Glands were homogenized in 30 µL of phosphate-buffered saline (PBS), and 10 µl of the homogenate was loaded onto a hemocytometer for counting under a light microscopy (Leica).

Dot plots of parasite count (oocysts and sporozoites) were generated in GraphPad Prism version 10. Mean and median intensities of infection were calculated for each group. Prevalence (percentage of infected mosquitoes) and intensity data from independent biological replicates were pooled prior to analysis to maintain balanced sample sizes across treatments. Infection intensity was compared between experimental and control groups using the two-tailed Mann-Whitney *U* test. Infection prevalence was compared using Fisher's exact test. Differences were considered statistically significant at $P < 0.05$. Raw data are listed in Dataset S1.

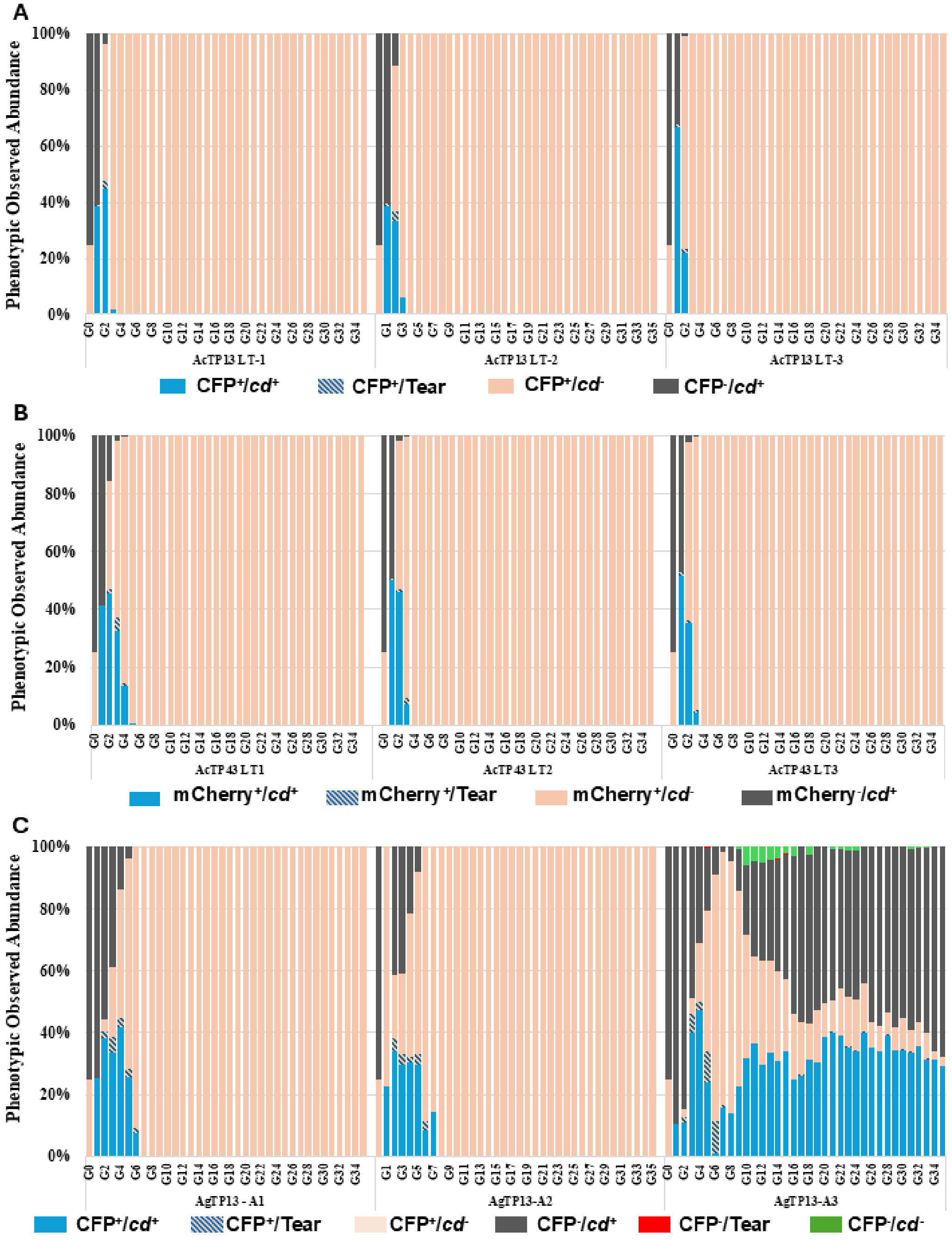

Figure S1. Distribution of phenotypes in long-term, discrete, non-overlapping generation cage trials. Percentage of total of each fluorescent and eye-color phenotype in each cage at each generation; AcTP13-LT1, LT2, LT3 (**A**), AcTP43-LT1, LT2, LT3 (**B**) and AgTP13-A1, A2, A3 (**C**). CFP$^+$/*cd*$^+$ mosquitoes are positive for the gene-drive system with wild-type eye color. CFP$^+$/Tear mosquitoes are positive for the gene-drive system with one or both eyes mosaic. CFP$^+$/*cd*$^-$ mosquitoes are positive for the gene-drive system with a *cardinal* eye phenotype. CFP$^-$/*cd*$^+$ mosquitoes are negative for the gene-drive system with a wild-type eye phenotype. CFP$^-$/*cd*$^-$ mosquitoes are negative for the gene-drive system with a *cardinal* eye phenotype. CFP$^-$/Tear mosquitoes are negative for the gene-drive system with one or both eyes mosaic. AgTP13 G0-13 data from Carballar-Lejarazú *et al.*[1] are used with permission.

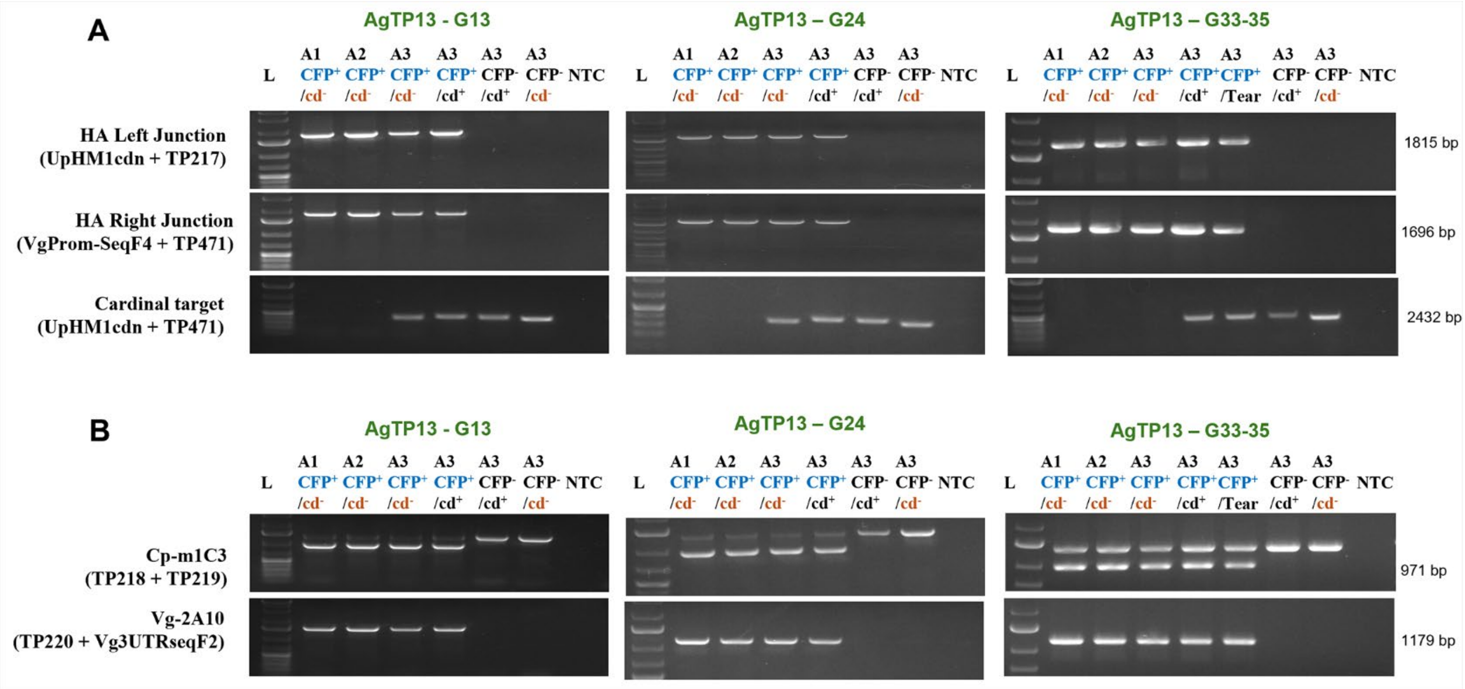

Figure S2. Drive stability validation of AgTP13 cage trials. (**A**) Gene amplification validation of drive system in AgTP13 cage trials at G13, G24 and G33-35. Left and right junction between the drive cassette and the *cd* homology arm were amplified to verify proper drive system insertion. Amplification of region of cd gene spanning the gRNA target site indicated the presence or absence of the drive insertion at the target site. (**B**) Amplification of effectors cassette using primers between 5' untranslated region (UTR) and 3'UTR for sequencing of the effectors m1C3 and m2A10.

a

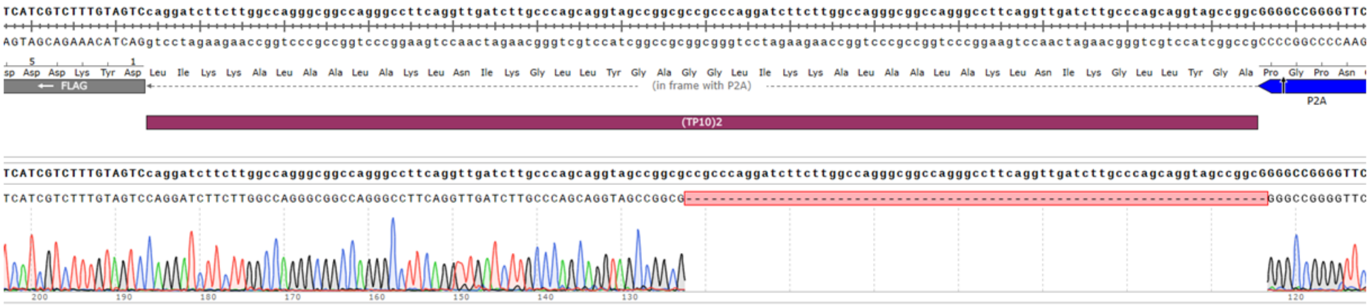

b

Generation	(TP10)2 allele frequency	TP10 allele frequency
G0	100% (100/100)	
G2	100% (50/50)	
G3	100% (102/102)	
G4	94% (94/100)	6% (6/100)
G6	93% (93/100)	7% (7/100)
G10	89% (89/100)	11% (11/100)
G18	77% (80/104)	23% (24/104)
G35	65% (68/104)	35% (36/104)

Figure S3. Mutation analysis of cage AcTP43 -LT3. (**a**) Sanger sequencing revealed a mutation in the MultiEff cassette of TP43 drive system that cause (TP10)2 dimer to lose a TP10 monomer. (**b**) Frequency of (TP10)2 dimer and TP10 mutated alleles at selective generations.

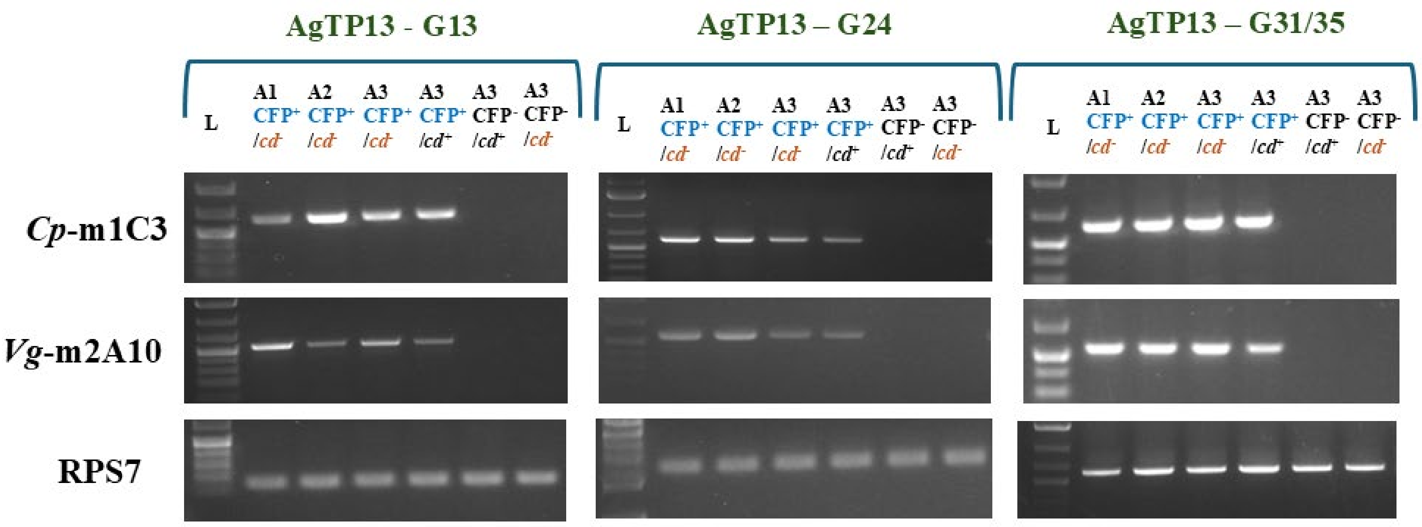

Figure S4. Effector expression of AgTP13 cage trials. Gene specific RT-PCR of m1C3 and m2A10 driven by *Cp* and *Vg* promoters, respectively, in AgTP13-A1, A2, A3 cages at selected generation. Female mosquitoes with each phenotype were collected from each cage at 24 H post blood meal and extracted for total RNA for RT-PCR templates. Ribosomal protein RPS7 was used as a control. L: molecular weight ladder.

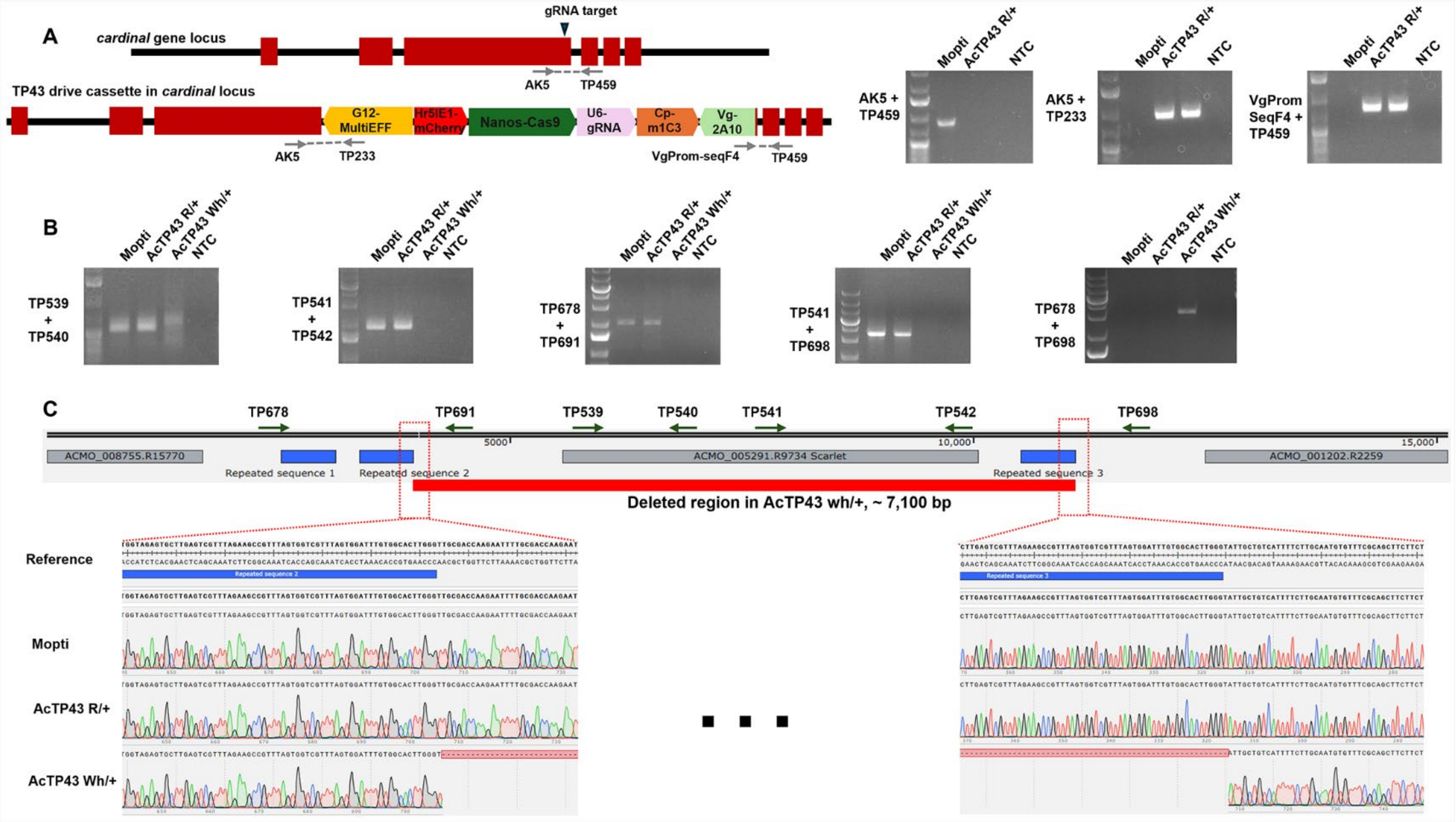

Figure S5. Molecular analysis of white-eyed mosquitoes recovered from the AcTP43 drive efficacy experiment. (**A**) The white-eyed (wh), mCherry-positive mosquito, AcTP43 wh/+, show disruption of the *cardinal* gene at the gRNA target (AK5+TP459) and correct integration of the TP43 cassette with the amplicon flanking the left (AK5+TP233) and right (VgPromSeqF4+TP459) junctions. The same is found in the red-eyed, mCherry-positive AcTP43 R/+ insects. (**B**) Gel electrophoresis of the PCR amplicons of the *scarlet* gene ACMO_005291 (VectorBase) and nearby regions showed that the AcTP43 wh/+ mosquitoes have lost the genomic region corresponding to the wild-type Mopti and AcTP43 R/+. *scarlet* gene (TP539+TP540; TP541+TP542). A ~2.0kb fragment of the AcTP43 wh/+ sequence surrounding the *scarlet* gene was amplified with the primer pairs, TP678 and TP698, which are spaced ~9.1kb apart in the wild-type undeleted genomic sequence. (**C**) Genome sequence alignments show that the AcTP43 wh/+ samples have an ~7.1 kb deletion (red bar) surrounding the *scarlet* gene with borders flanking by two repeat sequences (2 and 3, blue bars). Gray bar, genes; arrows show location of oligonucleotide primers with names listed underneath. NTC, non-template control.

Table S1. Long-term cage trial data AcTP13 cage LT1.

Gen	Total	Larvae CFP$^+$ (%)	Larvae CFP$^-$ (%)	Pupae and adults CFP$^+$						Pupae and adults CFP$^-$			
				WT (cd$^+$)		Tear		cd$^-$		WT (cd$^+$)		cd$^-$	
				Male	Female	Male	Female	Male	Female	Male	Female	Male	Female
G0	300	75 (25%)	225 (75%)					75		75	150		
G1	3841	250 (39.25%)	387 (60.75%)	127	120	1	2			213	174		
G2	8118	490 (96.5%)	18 (3.5%)	109	118	5	10	187	161	8	10		
G3	6784	669 (100%)		6	5		2	376	280				
G4	6092	687 (100%)						348	339				
G5	8854	597 (100%)						268	329				
G6	7570	615 (100%)						322	293				
G7	6941	611 (100%)						341	270				
G8	7288	638 (100%)						361	277				
G9	12617	613 (100%)						334	279				
G10	7445	625 (100%)						370	255				
G11	9917	660 (100%)						354	306				
G12	10071	665 (100%)						339	326				
G13	10433	689 (100%)						385	304				
G14	8918	600 (100%)						333	267				
G15	8818	615 (100%)						329	286				
G16	11646	628 (100%)						356	272				
G17	8081	690 (100%)						356	334				
G18	10714	642 (100%)						336	306				
G19	5749	655 (100%)						363	292				
G20	6764	691 (100%)						342	349				
G21	4982	652 (100%)						337	315				
G22	7595	600 (100%)						329	271				
G23	8252	711 (100%)						367	344				
G24	9173	609 (100%)						326	283				
G25	9257	600 (100%)						334	266				
G26	9315	651 (100%)						325	326				
G27	7831	697 (100%)						382	315				
G28	6402	626 (100%)						343	283				
G29	8102	615 (100%)						335	280				

G30	8432	600 (100%)						321	279				
G31	7389	602 (100%)						312	290				
G32	6236	601 (100%)						306	295				
G33	11740	626 (100%)						338	288				
G34	9694	639 (100%)						308	331				
G35	9770	608 (100%)						294	314				
**Total**	**291,131**	**21842**	**630**	**242**	**243**	**6**	**14**	**11432**	**10005**	**296**	**334**		

Abbreviations: CFP, cyan fluorescent protein (positive +, negative -); WT, wild-type eye color; cd, cardinal (pale red-eye, positive +, negative -); tear, mosaic eye.

Table S2. Long-term cage trial data AcTP13 cage LT2.

Gen	Total	Larvae CFP$^+$ (%)	Larvae CFP$^-$ (%)	Pupae and adults CFP$^+$						Pupae and adults CFP$^-$			
				WT (cd+)		Tear		cd-		WT (cd+)		cd-	
				Male	Female	Male	Female	Male	Female	Male	Female	Male	Female
G0	300	75 (25%)	225 (75%)					75		75	150		
G1	3712	274 (40.8%)	398 (59.2%)	139	129	4	2			210	188		
G2	7623	513 (88.6%)	66 (11.4%)	87	107	9	9	160	141	31	35		
G3	6499	687 (100%)		21	18			298	300				
G4	10150	625 (100%)						326	299				
G5	9551	600 (100%)						293	307				
G6	7398	662 (100%)						346	313				
G7	8030	604 (100%)						294	310				
G8	8039	618 (100%)						311	307				
G9	12496	658 (100%)						350	308				
G10	10167	634 (100%)						301	333				
G11	9428	693 (100%)						362	331				
G12	8188	622 (100%)						344	278				
G13	8393	740 (100%)						383	357				
G14	8764	600 (100%)						323	277				
G15	6301	673 (100%)						342	331				
G16	4597	600 (100%)						298	302				
G17	11354	648 (100%)						334	314				
G18	6632	649 (100%)						320	329				
G19	4595	661 (100%)						339	322				
G20	7139	611 (100%)						315	296				
G21	3611	610 (100%)						337	273				
G22	7700	606 (100%)						315	291				
G23	7459	726 (100%)						357	369				
G24	9521	630 (100%)						342	288				
G25	8248	608 (100%)						299	309				
G26	9956	610 (100%)						292	318				
G27	10084	603 (100%)						324	279				

G28	6752	610 (100%)						333	277					
G29	10440	602 (100%)						320	282					
G30	7986	601 (100%)						333	268					
G31	9413	600 (100%)						344	256					
G32	6451	605 (100%)						326	279					
G33	9580	600 (100%)						340	260					
G34	8361	617 (100%)						318	299					
G35	8300	602 (100%)						297	305					
**Total**	**283218**	**21677**	**689**	**247**	**254**	**13**	**11**	**10991**	**10108**	**316**	**373**			

Abbreviations: CFP, cyan fluorescent protein (positive +, negative -); WT, wild-type eye color; cd, cardinal (pale red-eye, positive +, negative -); tear, mosaic eye.

Table S3. Long-term cage trial data AcTP13 cage LT3.

Gen	Total	Larvae CFP^{+} (%)	Larvae CFP^{-} (%)	Pupae and adults CFP^{+}						Pupae and adults CFP^{-}			
				WT (cd^{+})		Tear		cd^{-}		WT (cd^{+})		cd^{-}	
				Male	Female	Male	Female	Male	Female	Male	Female	Male	Female
G0	300	75 (25%)	225 (75%)					75		75	150		
G1	2730	536 (67.59%)	257 (32.41%)	210	320	4	2			140	117		
G2	9700	581 (99.2%)	5 (0.8%)	64	65	1	6	215	230	2	3		
G3	7011	681 (100%)			1			372	308				
G4	10129	623 (100%)						345	278				
G5	10831	595 (100%)						290	305				
G6	7629	602 (100%)						338	264				
G7	7905	608 (100%)						295	313				
G8	7773	616 (100%)						339	277				
G9	8984	618 (100%)						332	286				
G10	9072	652 (100%)						316	336				
G11	7224	678 (100%)						349	329				
G12	7405	637 (100%)						324	313				
G13	9442	678 (100%)						354	324				
G14	6034	600 (100%)						343	257				
G15	6645	666 (100%)						328	338				
G16	9152	608 (100%)						288	320				
G17	7931	619 (100%)						335	284				
G18	9085	701 (100%)						375	326				
G19	4743	603 (100%)						305	298				
G20	6088	645 (100%)						339	306				
G21	3109	600 (100%)						322	278				
G22	7160	594 (100%)						299	295				
G23	7914	682 (100%)						361	321				
G24	8778	677 (100%)						355	322				
G25	8181	601 (100%)						293	308				
G26	9440	611 (100%)						356	255				
G27	6530	683 (100%)						342	341				

**G28**	**6656**	**617 (100%)**						**317**	**300**				
**G29**	**10182**	**600 (100%)**						**336**	**264**				
**G30**	**8721**	**602 (100%)**						**311**	**291**				
**G31**	**10352**	**641 (100%)**						**350**	**291**				
**G32**	**5516**	**637 (100%)**						**372**	**265**				
**G33**	**10810**	**614 (100%)**						**327**	**287**				
**G34**	**10625**	**607 (100%)**						**304**	**303**				
**G35**	**11521**	**627 (100%)**						**345**	**282**				
**Total**	**281308**	**22015**	**487**	**274**	**386**	**5**	**8**	**11247**	**10095**	**217**	**270**		

Abbreviations: CFP, cyan fluorescent protein (positive +, negative -); WT, wild-type eye color; cd, cardinal (pale red-eye, positive +, negative -); tear, mosaic eye.

Table S4. Long-term cage trial data AcTP43 cage LT1.

Gen	Total	Larvae mCherry$^+$ (%)	Larvae mCherry$^-$ (%)	Pupae and adults Cherry$^+$						Pupae and adults DSR$^-$			
				WT (cd$^+$)		Tear		cd$^-$		WT (cd+)		cd-	
				Male	Female	Male	Female	Male	Female	Male	Female	Male	Female
G0	300	75 (25%)	225 (75%)					75		75	150		
G1	4576	296 (41.3%)	420 (58.7%)	151	144		1			224	196		
G2	5814	548 (84.2%)	103 (15.8%)	125	171	6	4	100	142	52	51		
G3	6761	545 (98.4%)	9 (1.6%)	89	91	15	12	153	185	6	3		
G4	3820	633 (99.8%)	1 (0.2%)	45	41	4	3	260	280		1		
G5	3000	345 (100%)						153	190				
G6	5297	616 (100%)						301	315				
G7	5125	600 (100%)						289	311				
G8	6722	655 (100%)						316	339				
G9	4990	635 (100%)						311	324				
G10	7835	614 (100%)						302	312				
G11	5097	643 (100%)						318	325				
G12	7265	629 (100%)						303	326				
G13	4707	606 (100%)						283	323				
G14	3110	497 (100%)						254	243				
G15	4208	611 (100%)						310	301				
G16	5447	604 (100%)						277	327				
G17	3159	643 (100%)						301	342				
G18	4231	645 (100%)						309	336				
G19	3362	603 (100%)						306	297				
G20	5162	647 (100%)						316	331				
G21	3503	676 (100%)						322	354				
G22	6389	640 (100%)						326	314				
G23	6618	636 (100%)						302	334				
G24	5839	674 (100%)						337	337				
G25	4457	600 (100%)						314	286				
G26	5550	618 (100%)						284	334				
G27	6099	600 (100%)						280	320				

**G28**	**6678**	**604 (100%)**						**269**	**335**				
**G29**	**5597**	**613 (100%)**						**290**	**323**				
**G30**	**4635**	**600 (100%)**						**286**	**314**				
**G31**	**5918**	**605 (100%)**						**300**	**305**				
**G32**	**8450**	**627 (100%)**						**311**	**316**				
**G33**	**4913**	**600 (100%)**						**272**	**328**				
**G34**	**5163**	**642 (100%)**						**338**	**304**				
**G35**	**5014**	**605 (100%)**						**301**	**304**				
**Total**	**184811**	**21030**	**758**	**410**	**447**	**25**	**20**	**9769**	**10357**	**357**	**401**		

Abbreviations: Cherry fluorescent protein (positive +, negative -); WT, wild-type eye color; cd, cardinal (pale red-eye, positive +, negative-); tear, mosaic eye.

Table S5. Long-term cage trial data AcTP43 cage LT2.

Gen	Total	Larvae mCherry⁺ (%)	Larvae mCherry⁻ (%)	Pupae and adults Cherry$^+$						Pupae and adults DSR $^-$			
				WT (cd$^+$)		Tear		cd$^-$		WT (cd$^+$)		cd$^-$	
				Male	Female	Male	Female	Male	Female	Male	Female	Male	Female
G0	300	75 (25%)	225 (75%)					75		75	150		
G1	5654	307 (50.5%)	301 (49.5%)	148	157	1	1	0	0	163	138		
G2	6916	620 (98.3%)	11 (1.7%)	146	144	4	3	151	172	5	6		
G3	6715	586 (99.7%)	2 (0.3%)	22	19	9	6	247	283		2		
G4	3012	606 (100%)		1				284	321				
G5	2488	518 (100%)						247	271				
G6	5824	621 (100%)						298	323				
G7	5661	600 (100%)						292	308				
G8	5583	621 (100%)						293	328				
G9	6017	632 (100%)						299	333				
G10	7259	612 (100%)						309	303				
G11	2807	600 (100%)						294	306				
G12	5567	625 (100%)						295	330				
G13	6553	600 (100%)						262	338				
G14	6984	611 (100%)						327	284				
G15	4469	624 (100%)						316	308				
G16	5781	640 (100%)						295	345				
G17	5369	608 (100%)						276	332				
G18	6489	546 (100%)						245	301				
G19	4644	618 (100%)						314	304				
G20	6514	575 (100%)						262	313				
G21	3634	622 (100%)						312	310				
G22	5946	659 (100%)						359	300				
G23	6942	630 (100%)						311	319				
G24	5313	643 (100%)						342	301				
G25	7353	600 (100%)						295	305				
G26	6872	602 (100%)						268	334				
G27	9803	609 (100%)						303	306				

G28	7349	600 (100%)						301	299				
G29	6288	631 (100%)						340	291				
G30	5463	601 (100%)						287	314				
G31	6442	615 (100%)						292	323				
G32	4861	603 (100%)						313	290				
G33	7373	608 (100%)						298	310				
G34	4921	601 (100%)						320	281				
G35	5521	604 (100%)						337	267				
**Total**	**204687**	**21073**	**539**	**317**	**320**	**14**	**10**	**10059**	**10353**	**243**	**296**		

Abbreviations: Cherry fluorescent protein (positive +, negative -); WT, wild-type eye color; cd, cardinal (pale red-eye, positive +, negative-); tear, mosaic eye.

Table S6. Long-term cage trial data AcTP43 cage LT3.

Gen	Total	Larvae mCherry$^+$ (%)	Larvae mCherry$^-$ (%)	Pupae and adults Cherry$^+$						Pupae and adults DSR$^-$			
				WT (cd$^+$)		Tear		cd$^-$		WT (cd$^+$)		cd$^-$	
				Male	Female	Male	Female	Male	Female	Male	Female	Male	Female
G0	300	75 (25%)	225 (75%)					75		75	150		
G1	5767	374 (53.0%)	331 (47.0%)	204	159	5	6			175	156		
G2	6977	632 (97.7%)	15 (2.3%)	110	117	5	3	189	208	9	6		
G3	7755	603 (99.8%)	1 (0.2%)	13	14	1	3	243	329	1			
G4	3212	631 (100%)						282	349				
G5	5253	629 (100%)						313	316				
G6	5870	614 (100%)						321	293				
G7	3570	664 (100%)						309	355				
G8	8666	610 (100%)						320	290				
G9	6501	667 (100%)						342	325				
G10	7398	657 (100%)						325	332				
G11	5714	629 (100%)						322	307				
G12	9365	643 (100%)						296	347				
G13	6789	600 (100%)						288	312				
G14	5313	572 (100%)						275	297				
G15	3423	672 (100%)						337	335				
G16	4902	698 (100%)						367	331				
G17	5436	633 (100%)						302	331				
G18	8353	604 (100%)						264	340				
G19	4895	621 (100%)						311	310				
G20	8857	623 (100%)						274	349				
G21	4628	657 (100%)						294	363				
G22	7771	669 (100%)						346	323				
G23	6389	616 (100%)						295	321				
G24	6791	635 (100%)						325	310				
G25	5978	600 (100%)						306	294				
G26	7753	616 (100%)						302	314				
G27	7430	647 (100%)						324	323				

**G28**	**9504**	**602 (100%)**						**314**	**288**				
**G29**	**6827**	**616 (100%)**						**311**	**305**				
**G30**	**7930**	**600 (100%)**						**293**	**307**				
**G31**	**5840**	**627 (100%)**						**317**	**310**				
**G32**	**8190**	**600 (100%)**						**307**	**293**				
**G33**	**10696**	**616 (100%)**						**326**	**290**				
**G34**	**6758**	**601 (100%)**						**297**	**304**				
**G35**	**6583**	**609 (100%)**						**314**	**295**				
**Total**	**233384**	**21762**	**572**	**327**	**290**	**11**	**12**	**10426**	**10696**	**260**	**312**		

Abbreviations: Cherry fluorescent protein (positive +, negative -); WT, wild-type eye color; cd, cardinal (pale red-eye, positive +, negative-); tear, mosaic eye.

Table S7. Long-term cage trial data AgTP13 cage A1.

Gen	Total	Larvae CFP$^+$ (%)	Larvae CFP$^-$ (%)	Pupae and adults CFP$^+$						Pupae and adults CFP$^-$			
				WT (cd$^+$)		Tear		cd$^-$		WT (cd$^+$)		cd$^-$	
				Male	Female	Male	Female	Male	Female	Male	Female	Male	Female
G0	300	75 (25%)	225 (75%)					75		75	150		
G1	3016	155 (25.70%)	448 (74.30%)	67	87		1			201	247		
G2	2864	239 (44.26%)	301 (55.74%)	120	86	6	7	11	9	154	147		
G3	6014	382 (61.02%)	244 (38.98%)	113	96	11	21	76	65	131	113		
G4	2002	457 (86.23%)	73 (13.77%)	112	108	6	12	108	111	29	44		
G5	1749	565 (96.25%)	22 (3.75%)	73	79	4	9	189	211	11	11		
G6	2326	509 (100%)		19	20	4	5	206	255				
G7	956	483 (100%)						230	253				
G8	775	310 (100%)						165	145				
G9	1478	614 (100%)						308	306				
G10	1568	595 (100%)						289	306				
G11	2918	633 (100%)						333	300				
G12	5439	584 (100%)						268	316				
G13	3922	604 (100%)						299	305				
G14	1912	639						306	333				
G15	4659	624						321	303				
G16	1063	580						277	303				
G17	1271	610						267	343				
G18	1537	661						323	338				
G19	1117	617						301	316				
G20	605	315						234	158				
G21	1244	641						328	313				
G22	4024	656						315	344				
G23	4965	613						299	314				
G24	4465	600						318	282				
G25	6823	681						369	312				
G26	4433	607						306	301				
G27	5617	601						309	292				

G28	5959	622						321	301					
G29	5550	635						328	307					
G30	8023	709						369	340					
G31	7178	649						323	326					
G32	5366	608						291	317					
G33	4517	600						290	310					
G34	5507	606						370	336					
G35	7961	653						328	325					
**Total**	**129123**	**19732**	**1313**	**504**	**476**	**31**	**55**	**9450**	**9396**	**601**	**712**			

Abbreviations: CFP, cyan fluorescent protein (positive +, negative -); WT, wild-type eye color; cd, cardinal (pale red-eye, positive +, negative-); tear, mosaic eye. AgTP13 G0-13 data from Carballar-Lejarazú *et al.*[1] are used with permission.

Table S8. Long-term cage trial data AgTP13 cage A2.													
Gen	Total	Larvae CFP$^+$ (%)	Larvae CFP$^-$ (%)	Pupae and adults CFP$^+$						Pupae and adults CFP $^-$			
				WT (cd$^+$)		Tear		cd$^-$		WT (cd$^+$)		cd$^-$	
				Male	Female	Male	Female	Male	Female	Male	Female	Male	Female
G0	300	75 (25%)	225 (75%)					75		75	150		
G1	4239	131 (22.66%)	447 (77.34%)	75	55	1				233	214		
G2	3589	267 (58.81%)	187 (41.19%)	66	87	6	15	47	46	111	76		
G3	2992	292 (58.99%)	203 (41.01%)	62	84	10	7	59	70	95	108		
G4	2908	393 (78.60%)	107 (21.40%)	69	83	4	5	109	123	42	65		
G5	1928	604 (92.07%)	52 (7.93%)	95	98	13	12	176	210	30	22		
G6	3061	554 (99.82%)	1 (0.18%)	25	21	5	12	214	277	1			
G7	751	313 (100%)		20	25			123	145				
G8	1682	615 (100%)						288	327				
G9	789	438 (100%)						230	208				
G10	3296	606 (100%)						281	325				
G11	3782	673 (100%)						311	362				
G12	3534	650 (100%)						292	358				
G13	3340	631 (100%)						299	332				
G14	3526	641 (100%)						300	341				
G15	3609	607 (100%)						292	315				
G16	1359	604 (100%)						299	305				
G17	885	380 (100%)						166	214				
G18	1214	627 (100%)						322	305				
G19	1817	636 (100%)						313	323				
G20	1983	687 (100%)						337	350				
G21	2063	642 (100%)						317	325				
G22	4312	625 (100%)						302	323				
G23	3619	618 (100%)						315	303				
G24	3322	600 (100%)						305	295				
G25	5845	621 (100%)						316	305				
G26	2250	600 (100%)						304	296				
G27	3377	632 (100%)						323	309				

**G28**	**4822**	**615 (100%)**						**307**	**308**				
**G29**	**4353**	**679**						**342**	**337**				
**G30**	**4193**	**653**						**333**	**320**				
**G31**	**4726**	**680**						**323**	**357**				
**G32**	**3881**	**600**						**316**	**284**				
**G33**	**3793**	**600**						**317**	**283**				
**G34**	**3909**	**633**						**320**	**313**				
**G35**	**4397**	**644**						**351**	**293**				
**Total**	**109446**	**19866**	**1222**	**412**	**453**	**39**	**51**	**9324**	**9587**	**587**	**635**		

Abbreviations: CFP, cyan fluorescent protein (positive +, negative -); WT, wild-type eye color; cd, cardinal (pale red-eye, positive +, negative-); tear, mosaic eye. AgTP13 G0-13 data from Carballar-Lejarazú *et al.*[1] are used with permission.

Table S9. Long-term cage trial data AgTP13 cage A3.

Gen	Total	Larvae CFP+ (%)	Larvae CFP- (%)	Pupae and adults CFP+						Pupae and adults CFP -					
				WT (cd^+)		Tear		cd^-		WT (cd^+)		Tear		cd^-	
				Male	Female	Male	Female	Male	Female	Male	Female	Male	Female	Male	Female
G0	300	75 (25%)	225 (75%)					75		75	150				
G1	2260	65 (10.55%)	551 (89.45%)	31	34					266	285				
G2	5348	85 (15.21%)	474 (84.79%)	30	31	5	6	6	7	224	250				
G3	2621	282 (51.27%)	268 (48.73%)	107	113	12	20	15	15	116	152				
G4	7151	411 (69.08%)	184 (30.92%)	130	151	6	9	62	53	85	99				
G5	1391	527 (79.25%)	138 (20.75%)	74	85	32	34	152	150	64	71	3			
G6	2347	538 (95.05%)	28 (4.95%)	115	140	8	25	111	139	12	16				
G7	3449	452 (98.26%)	8 (1.74%)	38	34	2	2	172	204	4	4				
G8	1421	593 (95.49%)	28 (4.51%)	34	52	1		232	274	15	13				
G9	2062	529 (86.02%)	86 (13.98%)	71	72			195	191	44	38			1	3
G10	3736	425 (71.43%)	170 (28.57%)	85	103			111	126	69	67			15	19
G11	2812	446 (64.73%)	243 (35.27%)	126	125			104	91	110	101			16	16
G12	4540	394 (63.75%)	224 (36.25%)	93	96			87	118	101	91		4	18	13
G13	4226	391 (63.17%)	228 (36.83%)	102	105			95	89	102	100			13	13
G14	4773	378 (59.90%)	253 (40.10%)	99	95			79	105	114	113	1		12	13
G15	3480	356 (57.33%)	265 (42.67%)	89	122			57	88	133	118		1	8	5
G16	2699	276 (46.00%)	324 (54.00%)	65	83			48	80	141	166			5	12
G17	2283	282 (43.45%)	367 (56.55%)	77	93		1	46	65	146	221				
G18	2954	261 (42.93%)	347 (57.07%)	86	104			39	32	151	182			8	6

G19	1918	293 (47349%)	324 (52.51%)	91	97			51	54	150	174				
G20	2321	318 (49.53%)	324 (50.47%)	130	118			34	36	138	186				
G21	3374	299 (50.25%)	296 (49.75%)	119	119	1	1	22	37	135	158			3	
G22	5642	349 (54.36%)	293 (45.64%)	118	132	1		48	50	130	157			2	4
G23	4471	331 (53.13%)	292 (46.87%)	115	116	1	1	49	49	154	132			3	3
G24	4183	304 (50.75%)	295 (49.25%)	99	105		1	50	50	133	154			4	4
G25	5422	340 (56.01%)	267 (43.99%)	126	117	1	2	44	50	121	146				
G26	5827	261 (43.50%)	339 (56.50%)	107	103			23	28	154	185				
G27	6717	290 (42.15%)	398 (57.85%)	104	129			29	28	202	196				
G28	7736	286 (46.58%)	328 (53.42%)	126	115	1	1	18	25	171	157				
G29	6834	254 (41.71%)	355 (58.29%)	94	114			22	24	170	185				
G30	9983	306 (44.87%)	376 (55.13%)	108	126	2		35	35	177	199				
G31	9090	261 (40.7%)	379 (59.22%)	98	115	1	2	25	20	180	195				4
G32	7834	273 (43.47%)	355 (56.53%)	94	129	1	1	23	25	160	194			1	
G33	6292	248 (39.87%)	374 (60.13%)	107	88	2	1	22	28	180	193			1	
G34	3059	226 (34.09%)	437 (65.91%)	111	97			7	11	202	235				
G35	5756	203 (32.12%)	429 (67.88%)	91	93		1	9	9	214	215				
**Total**	**156312**	**11608**	**10272**	**3290**	**3551**	**77**	**108**	**2197**	**2386**	**4743**	**5298**	**4**	**5**	**110**	**115**

Abbreviations: CFP, cyan fluorescent protein (positive +, negative -); WT, wild-type eye color; cd, cardinal (pale red-eye, positive +, negative-); tear, mosaic eye. AgTP13 G0-13 data from Carballar-Lejarazú *et al.*[1] are used with permission.

**Table S10. List of oligonucleotides and primers.**		
**Name of primer**	**Sequence (5'– 3')**	**Reference**
CO30	TCCACATCCGAGTTATCCGGGT	This study
off-1-EZ-F	ACACTCTTTCCCTACACGACGCTCTTCCGATCTACAAGTGGATGATATCCC TGAACA	Carballar-Lejarazú *et al.*[3]
off-1-EZ-R	GACTGGAGTTCAGACGTGTGCTCTTCCGATCTAGTGCCTGTCTCGGCTGCT GTATG	Carballar-Lejarazú *et al.*[3]
RPS7-F	GACGGATCCCAGCTGATAAA	Carballar-Lejarazú *et al.*[1]
RPS7-R	GTTCTCTGGGAATTCGAACG	Carballar-Lejarazú *et al.*[1]
RT-MultiEff-F	GGCATCGGCGCCGTGCTGAAG	Dong *et al.*[7]
RT-MultiEff-R2	CTTGTCGTCATCGTCTTTGTAGTC	Carballar-Lejarazú *et al.*[2]
TP179	GAAAGTCACCCTCGTCCTACG	Carballar-Lejarazú *et al.*[1]
TP217	GCTTGTTTGAATTGAATTGTCGC	Carballar-Lejarazú *et al.*[1]
TP218	GTCGATTGAACATGGTGCGAT	Carballar-Lejarazú *et al.*[1]
TP219	AAGCAGCGCTAAGCCACC	Carballar-Lejarazú *et al.*[1]
TP220	CGGCGGTCATTACACTATTC	Carballar-Lejarazú *et al.*[1]
TP233	CTAAATCGAACGAACACCAATGG	This study
TP236	TACGTTTAAGACGTACGCCATGAACTG	This study
TP237	CTTCAGCTCCAGTTTGGTACCCG	This study
TP240	ACGCTTAATTTCCAGCTTCGTGC	This study
TP241	GGATTAACACGATCACGGAAGAACC	This study
TP400	CAGCTTCGAATATGATTTCAATG	This study
TP401	CATTCCCAAGCCTACTACAGC	This study
TP456	CTCGATCGGTCTGGATATCG	This study
TP539	CAAAACCCAAAACCGAAACCGAACCGCCGG	This study
TP540	TGTAGCTCCCGAACCCCTCCCACACGCAGC	This study
TP541	CCCCTGAATTCTTCCCCTGCTCGCACAACTCATCACTC	This study
TP542	CCAAACAAAAGCCGAGTACATGGTGAGAATTGCCCGG	This study
TP678	CTGCACGAATCGTATAAAATAAATGATAAAGTG	This study
TP691	CACACAAACATGGAGAATGATTTATTCTAC	This study
TP698	CTACTGCCGAGTGACTCCTAACCCAACGACCAC	This study
upHM1cdn-F	CAAACTCGGCGTACGTGAT	Carballar-Lejarazú *et al.*[3]
Vg3UTR-seqF2	GAATTACAACTAGGCTCGAGTTG	Carballar-Lejarazú *et al.*[1]
VgProm-seqF4	GAAGCCATACCATGAGTCCG	Carballar-Lejarazú *et al.*[1]

Table S11. Summary of molecular genotype analyses of AgTP13-A3 exceptional phenotype individuals.

Phenotype	Genotype	G6	G7	G8	G9	G10	G11	G12	G13	G18	G24	G33-35
CFP^{+}/cd^{+}	D/+	14/20 (70%)	2/20 (10%)	1/20 (5%)								
	D/R	6/20 (30%)	18/20 (90%)	19/20 (95%)	20/20 (100%)	19/20 (100%)	17/17 (100%)	18/18 (100%)	20/20 (100%)	20/20 (100%)	20/20 (100%)	30/30 (100%)
CFP^{-}/cd^{+}	+/+	21/23 (91%)										
	+/r	2/23 (9%)										
	+/R		2/6 (33%)	4/23 (17%)								
	R/R		4/6 (67%)	11/23 (48%)	11/16 (69%)	14/17 (82%)	5/17 (29%)	12/19 (63%)	13/18 (72%)	18/20 (900%)	20/20 (100%)	30/30 (100%)
	R/r			8/23 (35%)	5/16 (31%)	3/17 (18%)	12/17 (71%)	7/19 (37%)	5/18 (28%)	2/20 (10%)		
CFP^{-}/cd^{-}	r/r				3/3 (100%)	21/21 (100%)	19/19 (100%)	15/15 (100%)	17/17 (100%)	10/10 (100%)	1/1 (100%)	1/1 (100%)

Genotype: +, wild-type allele; R, In-frame Non-Homologous End Joining (NHEJ) mutated allele; r, out-of-frame NHEJ mutation; D, drive allele.

Table S12. Sequences of NHEJ alleles from analyzed AgTP13-A3 mosquitoes with exceptional phenotypes.

Gen	GATGAGTCGTCACCCGAGTGGAACGGTACGGCGGTTAGCGACGATGCCAAGGCGGCCGCCATAGCGGATGGCG	Frame
G6	GATGAGTCGTCACCCGAGTGGAACGGTACGGCGGTTAGCGACGAT-----------CGCCATAGCGGATGGCG	Out
G7	GATGAGTCGTCACCCGAGTGGAACGGTACGGCGGTTAGCGACGATGCCAtcgAGGCGGCCGCCATAGCGGATGGCG	In
G8	GATGAGTCGTCACCCGAGTGGAACGGTACGGCGGTTAGCGACGATGCCAtcgAGGCGGCCGCCATAGCGGATGGCG	In
	GATGAGTCGTCACCCGAGTGGAACGGTACGGCGGTTAGCGACGATGCCAccgcgttcgtcacccgagtggacgAGGCGG CCGCCATAGCGGATGGCG	In
	GATGAGTCGTCACCCGAGTGGAA-----------------------------CGGCCGCCATAGCGGATGGCG	Out
G9	GATGAGTCGTCACCCGAGTGGAACGGTACGGCGGTTAGCGACGATGCCAtcgAGGCGGCCGCCATAGCGGATGGCG	In
	GATGAGTCGTCACCCGAGTGGAA-----------------------------CGGCCGCCATAGCGGATGGCG	Out
G10	GATGAGTCGTCACCCGAGTGGAACGGTACGGCGGTTAGCGACGATGCCAtcgAGGCGGCCGCCATAGCGGATGGCG	In
	GATGAGTCGTCACCCGAGTGGAACGGTACGGCGGTTAGCGACGATGCCAccgcgttcgtcacccgagtggacgAGGCGG CCGCCATAGCGGATGGCG	In
	GATGAGTCGTCACCCGAGTGGAA-----------------------------CGGCCGCCATAGCGGATGGCG	Out
G11	GATGAGTCGTCACCCGAGTGGAACGGTACGGCGGTTAGCGACGATGCCAtcgAGGCGGCCGCCATAGCGGATGGCG	In
	GATGAGTCGTCACCCGAGTGGAACGGTACGGCGGTTAGCGACGATGCCAccgcgttcgtcacccgagtggacgAGGCGG CCGCCATAGCGGATGGCG	In
	GATGAGTCGTCACCCGAGTGGAA-----------------------------CGGCCGCCATAGCGGATGGCG	Out
G12	GATGAGTCGTCACCCGAGTGGAACGGTACGGCGGTTAGCGACGATGCCAtcgAGGCGGCCGCCATAGCGGATGGCG	In
	GATGAGTCGTCACCCGAGTGGAACGGTACGGCGGTTAGCGACGATGCCAccgcgttcgtcacccgagtggacgAGGCGG CCGCCATAGCGGATGGCG	In
	GATGAGTCGTCACCCGAGTGGAA-----------------------------CGGCCGCCATAGCGGATGGCG	Out
G13	GATGAGTCGTCACCCGAGTGGAACGGTACGGCGGTTAGCGACGATGCCAtcgAGGCGGCCGCCATAGCGGATGGCG	In
	GATGAGTCGTCACCCGAGTGGAACGGTACGGCGGTTAGCGACGATGCCAccgcgttcgtcacccgagtggacgAGGCGG CCGCCATAGCGGATGGCG	In
	GATGAGTCGTCACCCGAGTGGAA-----------------------------CGGCCGCCATAGCGGATGGCG	Out
G18	GATGAGTCGTCACCCGAGTGGAACGGTACGGCGGTTAGCGACGATGCCAtcgAGGCGGCCGCCATAGCGGATGGCG	In
	GATGAGTCGTCACCCGAGTGGAA-----------------------------CGGCCGCCATAGCGGATGGCG	Out
G24	GATGAGTCGTCACCCGAGTGGAACGGTACGGCGGTTAGCGACGATGCCAtcgAGGCGGCCGCCATAGCGGATGGCG	In
	GATGAGTCGTCACCCGAGTGGAA-----------------------------CGGCCGCCATAGCGGATGGCG	Out
G33-35	GATGAGTCGTCACCCGAGTGGAACGGTACGGCGGTTAGCGACGATGCCAtcgAGGCGGCCGCCATAGCGGATGGCG	In
	GATGAGTCGTCACCCGAGTGGAA-----------------------------CGGCCGCCATAGCGGATGGCG	Out

gRNA target site; PAM; insertion; deletion; Frame: In, in-frame; Out, frame-shift.

Table S13. Off-target 1 indel detection by NGS.				
**Samples**	**Reads**	**Indel%**	**Reads**	**Indel%**
**Time zero**				
AcTP13 CFP^{+}/cd^{-}	51,340	0.02		
AcTP43 $mCherry^{+}/cd^{-}$	41,573	0.03		
WT-Mopti			31,508	0.02
**AcTP13 Year 1**				
AcTP13 – LT1 CFP^{+}/cd^{-}	185,865	0.19		
AcTP13 – LT2 CFP^{+}/cd^{-}	144,761	0.2		
AcTP13 – LT3 CFP^{+}/cd^{-}	137,570	0.2		
WT-Mopti			210,848	0.19
**AcTP13 Year 2**				
AcTP13 – LT1 CFP^{+}/cd^{-}	267,390	0.14		
AcTP13 – LT2 CFP^{+}/cd^{-}	247,676	0.14		
AcTP13 – LT3 CFP^{+}/cd^{-}	228,399	0.15		
WT-Mopti			222,246	0.15
**AcTP43 Year 1**				
AcTP43 – LT1 $mCherry^{+}/cd^{-}$	311,650	0.56		
AcTP43 – LT2 $mCherry^{+}/cd^{-}$	315,584	0.6		
AcTP43 – LT3 $mCherry^{+}/cd^{-}$	179,582	0.5		
WT-Mopti			200,730	0.5
**AcTP43 Year 2**				
AcTP43 – LT1 $mCherry^{+}/cd^{-}$	283,659	0.12		
AcTP43 – LT2 $mCherry^{+}/cd^{-}$	309,290	0.13		
AcTP43 – LT3 $mCherry^{+}/cd^{-}$	291,217	0.15		
WT-Mopti			191,321	0.14
**AgTP13 - G13**				
A1 CFP^{+}/cd^{-}	583,741	0.18		
A2 CFP^{+}/cd^{-}	421,120	0.15		
A3 CFP^{+}/cd^{-}	362,800	0.17		
A3 CFP^{+}/cd^{+}	352,322	0.17		
A3 CFP^{-}/cd^{+}	441,943	0.17		
A3 CFP^{-}/cd^{-}	540,778	0.18		
WT – X1			317,732	0.17
**AgTP13 – G24**				
A1 CFP^{+}/cd^{-}	164,683	0.12		
A2 CFP^{+}/cd^{-}	94,743	0.12		
A3 CFP^{+}/cd^{-}	223,094	0.11		
A3 CFP^{+}/cd^{+}	254,401	0.1		
A3 CFP^{-}/cd^{+}	277,806	0.13		
A3 CFP^{-}/cd^{-}	204,208	0.11		
WT – X1	187,521	0.11	317,732	0.17
**AgTP13 – G33-35**				
A1 CFP^{+}/cd^{-}	354,495	0.12		
A2 CFP^{+}/cd^{-}	354,979	0.13		
A3 CFP^{+}/cd^{-}	333,474	0.12		
A3 CFP^{+}/cd^{+}	320,045	0.14		
A3 CFP^{-}/cd^{+}	267,549	0.11		
A3 CFP^{-}/cd^{-}	294,912	0.11		
WT – X1			288,933	0.14
**Total/ Average percent**	**8,842,649**	**0.1740625**	**1,650,839**	**0.1775**

Table S14. White-eye phenotypes recovered from AcTP43 long term cage trial drive efficiency experiments.

**Time zero**				
**Generation**	**Male-founder lineage[1]**		**Female-founder lineage[1]**	
**F_1**	♂ 0% (0/2352)		♀ 0% (2314/2323)	
**F_2**	♂ 0% (0/1118)	♀ 0% (0/1362)	♂ 0.07% (1/1413)	♀ 0% (0/1608)
**Year 1**				
**Generation**	**Male-founder lineage[1]**		**Female-founder lineage[1]**	
**F_1**	♂ 0% (0/2464)		♀ 0% (0/2345)	
**F_2**	♂ 0% (0/1609)	♀ 0% (0/1930)	♂ 0.43% (8/1854)	♀ 0% (0/2043)
**Year 2**				
**Generation**	**Male-founder lineage[1]**		**Female-founder lineage[1]**	
**F_1**	♂ 0% (0/2350)		♀ 0% (0/2551)	
**F_2**	♂ 0% (0/2117)	♀ 0% (0/1996)	♂ 0% (0/2028)	♀ 0% (0/2112)

[1]Symbols, ♂ and ♀, refer to the sex of the gene-drive parents and progeny.

**Table S15. Inheritance of white-eye phenotype recovered from AcTP43 long-term cage trial drive efficiency experiments.**															
**Parental outcross: 1 mCherry^{+}/white-eye ♂ x 20 WT-Mopti (mCherry^{-}/*cd*$^{+}$) ♀**															
**F1 progeny phenotypes[1]**															
**mCherry^{+}**								**mCherry^{-}**							
***cd*$^{+}$**		***cd*$^{-}$**		**white-eye**		**tear**		***cd*$^{+}$**		***cd*$^{-}$**		**white-eye**		**tear**	
♂	♀	♂	♀	♂	♀	♂	♀	♂	♀	♂	♀	♂	♀	♂	♀
40		0		0		0		0		0		0		0	
**F1 outcross: mCherry^{+}/*cd*$^{+}$ ♀ x 20 WT-Mopti (mCherry^{-}/*cd*$^{+}$) ♂**															
**F2 progeny phenotypes**															
141	229	0	1	190	0	0	2	2	3	0	0	0	0	0	0
**F2 intercross: mCherry^{+}/ white-eye ♂ x mCherry^{+}/ *cd*$^{+}$♀**															
**F3 progeny phenotypes**															
15	11	124	199	55	119	0	0	0	0	0	0	0	0	0	0

[1]F1 progeny were not scored for sex.
Abbreviations: mCherry, red fluorescent protein (positive +, negative -); *cd*$^{+}$, wild-type eye color; *cd*$^{-}$, cardinal (pale red-eye, positive +, negative -); tear, mosaic eye.

**Table S16. Sequences of genomic repeats 2 and 3 and the junction of the *scarlet* genome deletion.**	
**Sequence[1]**	
**Repeat 2[2]**	**CCCAAGCGCCACA**GATTAAGCTTAAACGACTGGAAAATTGAATATCCATAGAAAAAAA**A**GAAAGCTCCA<br>CAGCTTCATTGGTTTGATCAATAGATGGCGTATTA**C**AACT**C**AT**C**ATTATATTGCTATCCTTTTCTTGCA<br>ATGTGTTTCGACAAGTTTCATCTTATCATGA**T**CTATATAGCCTTCTAACTTTCTGAGCAAAAATCTATA<br>TGAATGGTCGTG**T**GGTCAGATACGTGGGTATCAACACCAACGACCATTACT**T**AAATCTCACTTGCTTCA<br>GTGCTGGTGGTT**A**CC**A**TTGGAGTTTAGTATTTAAACAAT**T**GTATCGCCGTTCTAGTTCTAGCGATGCAT<br>AACTTCAATGCGAAACCATACAACAG**C**ACAGAGGAAATGAGAAAGCTCT**A**CTCCAAGCGATGAAGCTCC<br>ACTGGTTGGGGTAT**T**CCAC**A**AACAGAGAGCGCCACCAGCTTTTT**C**GCTATTTTTAGAATGCATGAGTCG<br>TTTGCCGTCTGCTAAACGAAGTCTTTCTATATTCCGTTT**T**GGTAGAG**TG**CTTGAGTCGTTTAGAAGCCG<br>TTTAGTGGTCGTTTAGTGGATT**TGTGGCACTTGGG**
**Repeat 3[3]**	**CCCAAGTGCCACA**GATTAAGCTTAAACGACTGGAAAATTGAATATCCATAGAAAAAAA**T**GAAAGCTCCA<br>CAGCTTCATTGGTTTGATCAATAGATGGCGTATTA**A**AACT**G**AT**T**ATTATATTGCTATCCTTTTCTTGCA<br>ATGTGTTTCGACAAGTTTCATCTTATCATGACCTATATAGCCTTCTAACTTTCTGAGCAAAAATCTATA<br>TGAATGGTCGTG**A**GGTCAGATACGTGGGTATCAACACCAACGACCATTACT**C**AAATCTCACTTGCTTCA<br>GTGCTGGTGGTT**T**CC**G**TTGGAGTTTAGTATTTAAACAAT**C**GTATCGCCGTTCTAGTTCTAGCGATGCAT<br>AACTTCAATGCGAAACCATACAACAG**T**ACAGAGGAAATGAGAAAGCTCT**C**CTCCAAGCGATGAAGCTCA<br>ACTGGTTGGGGTAT**C**CCAC**C**AACAGAGAGCGCCACCAGCTTTTT**T**GCTATTTTTAGAATGCATGAGTCG<br>TTTGCCGTCTGCTAAACGAAGTCTTTCTATATTCCGTTT**A**GGTAGAG**AA**CTTGAGTCGTTTAGAAGCCG<br>TTTAGTGGTCGTTTAGTGGATT**TGTGGCACTTGGG**
**AcTP43 *scarlet*Δ[4]**	**CCCAAGCGCCACA**GATTAAGCTTAAACGACTGGAAAATTGAATATCCATAGAAAAAAA**A**GAAAGCTCCA<br>CAGCTTCATTGGTTTGATCAATAGATGGCGTATTA**A**AACT**G**AT**T**ATTATATTGCTATCCTTTTCTTGCA<br>ATGTGTTTCGACAAGTTTCATCTTATCATGA**C**CTATATAGCCTTCTAACTTTCTGAGCAAAAATCTATA<br>TGAATGGTCGTG**T**GGTCAGATACGTGGGTATCAACACCAACGACCATTACT**T**AAATCTCACTTGCTTCA<br>GTGCTGGTGGTT**A**CC**A**TTGGAGTTTAGTATTTAAACAATTGTATCGCCGTTCTAGTTCTAGCGATGCAT<br>AACTTCAATGCGAAACCATACAACAG**C**ACAGAGGAAATGAGAAAGCTCT**A**CTCCAAGCGATGAAGCTCC<br>ACTGGTTGGGGTAT**T**CCAC**A**AACAGAGAGCGCCACCAGCTTTTTCGCTATTTTTAGAATGCATGAGTCG<br>TTTGCCGTCTGCTAAACGAAGTCTTTCTATATTCCGTTT**T**GGTAGAG**TG**CTTGAGTCGTTTAGAAGCCG<br>TTTAGTGGTCGTTTAGTGGATT**TGTGGCACTTGGG**

[1]Inverted repeat sequences are bolded in black.
[2]Repeat 2 single nucleotide polymorphisms (SNPs) are bolded in red.
[3]Repeat 3 SNPs are bolded in blue.
[4]Sequence of the deletion region surrounding the *scarlet* locus. The underlined sequence has the Repeat 3 SNPs.

Table S17. AcTP13 long term cage trial drive efficiency experiment Time zero.

Generation	Male-founder lineage drive efficiency[1,2]		Female-founder lineage drive efficiency[1,2]	
F_1	♂ 99.9% (2322/2323)		♀ 99.2% (2815/2838)	
F_2	♂ 99.3% (1585/1596)	♀ 97.8% (1479/1512)	♂ 99.7% (1597/1602)	♀ 96.9% (1808/1865)
**Generation**	**Male-founder lineage *cardinal* eye[3]**		**Female-founder lineage *cardinal* eye[3]**	
F_1	♂ 0% (0/2323)		♀ 0.2% (7/2838)	
F_2	♂ 0% (0/1596)	♀ 0.3% (5/1512)	♂ 0% (0/1602)	♀ 0.05% (1/1865)
**Generation**	**Male lineage tear eye[4]**		**Female lineage tear eye[4]**	
F_1	♂ 0.5% (11/2323)		♀ 5.2% (148/2838)	
F_2	♂ 0.6% (9/1596)	♀ 4.4% (67/1512)	♂ 0.4% (6/1602)	♀ 5.5% (102/1865)

[1]Symbols refer to the sex of the gene-drive parents: ♂ male gene-drive parents; ♀ female gene-drive parents.
[2]Drive efficiency was determined by calculating the percentage of progeny expressing the CFP marker gene out of the total originating from the mating event.
[3]Eye phenotype was determined by calculating the percentage of heteroallelic progeny containing one copy of the drive element (CFP^+) and one resistant mutant *cardinal* (*cd*$^-$) allele out of the total originating from the mating event.
[4]Eye phenotype was determined by calculating the percentage of progeny showing the tear phenotype out of the total originating from the mating event.

Table S18. AcTP43 long term cage trial drive efficiency experiment Time zero.

Generation	Male-founder lineage drive efficiency[1,2]		Female-founder lineage drive efficiency[1,2]	
F_1	♂ 100% (2352/2352)		♀ 99.7% (2314/2323)	
F_2	♂ 99.9% (1117/1118)	♀ 98.9% (1347/1362)	♂ 99.5% (1406/1413)	♀ 95.5% (1535/1608)
**Generation**	**Male-founder lineage *cardinal* eye[3]**		**Female-founder lineage *cardinal* eye[3]**	
F_1	♂ 0% (0/2352)		♀ 0.9% (20/2323)	
F_2	♂ 0% (0/1118)	♀ 0.1% (2/1362)	♂ 0% (0/1413)	♀ 0.06% (1/1608)
**Generation**	**Male lineage tear eye[4]**		**Female lineage tear eye[4]**	
F_1	♂ 1.4% (33/2352)		♀ 13.1% (305/2323)	
F_2	♂ 0.3% (3/1118)	♀ 4.3% (58/1362)	♂ 0.6% (9/1413)	♀ 2.7% (44/1608)

[1]Symbols refer to the sex of the gene-drive parents: ♂ male gene-drive parents; ♀ female gene-drive parents.
[2]Drive efficiency was determined by calculating the percentage of progeny expressing the Cherry marker gene out of the total originating from the mating event.
[3]Eye phenotype was determined by calculating the percentage of heteroallelic progeny containing one copy of the drive element (Cherry$^+$) and one resistant mutant *cardinal* (*cd*$^-$) allele out of the total originating from the mating event.
[4]Eye phenotype was determined by calculating the percentage of progeny showing the tear phenotype out of the total originating from the mating event.

Table S19. AcTP13 long term cage trial drive efficiency experiment Year 1.

Generation	Male-founder lineage drive efficiency[1,2]		Female-founder lineage drive efficiency[1,2]	
F_1	♂ 100% (2057/2057)		♀ 99.8% (1697/1700)	
F_2	♂ 100% (1227/1227)	♀ 99.2% (1365/1376)	♂ 98.2% (1281/1305)	♀ 98.0% (1500/1531)
**Generation**	**Male-founder lineage *cardinal* eye[3]**		**Female-founder lineage *cardinal* eye[3]**	
F_1	♂ 0% (0/2057)		♀ 0.6% (10/1700)	
F_2	♂ 0% (0/1227)	♀ 0.2% (3/1376)	♂ 0% (0/1305)	♀ 0.3% (4/1531)
**Generation**	**Male lineage tear eye[4]**		**Female lineage tear eye[4]**	
F_1	♂ 0.7% (15/2057)		♀ 5.4% (92/1700)	
F_2	♂ 0.9% (11/1227)	♀ 10.0% (137/1376)	♂ 0.9% (12/1305)	♀ 7.6% (117/1531)

[1]Symbols refer to the sex of the gene-drive parents: ♂ male gene-drive parents; ♀ female gene-drive parents.
[2]Drive efficiency was determined by calculating the percentage of progeny expressing the CFP marker gene out of the total originating from the mating event.
[3]Eye phenotype was determined by calculating the percentage of heteroallelic progeny containing one copy of the drive element (CFP^+) and one resistant mutant *cardinal* (*cd*$^-$) allele out of the total originating from the mating event.
[4]Eye phenotype was determined by calculating the percentage of progeny showing the tear phenotype out of the total originating from the mating event.

Table S20. AcTP43 long term cage trial drive efficiency experiment Year 1.

Generation	Male-founder lineage drive efficiency[1,2]		Female-founder lineage drive efficiency[1,2]	
F_1	♂ 100% (2464/2464)		♀ 99.9% (2343/2345)	
F_2	♂ 99.9% (1608/1609)	♀ 99.4% (1918/1930)	♂ 99.8% (1851/1854)	♀ 97.7% (1997/2043)
**Generation**	**Male-founder lineage *cardinal* eye[3]**		**Female-founder lineage *cardinal* eye[3]**	
F_1	♂ 0% (0/2464)		♀ 0.5% (12/2345)	
F_2	♂ 0% (0/1609)	♀ 0% (0/1930)	♂ 0% (0/1854)	♀ 0.2% (5/2043)
**Generation**	**Male lineage tear eye[4]**		**Female lineage tear eye[4]**	
F_1	♂ 0.5% (12/2464)		♀ 10.0% (234/2345)	
F_2	♂ 0.2% (4/1609)	♀ 2.1% (40/1930)	♂ 0.5% (10/1854)	♀ 2.8% (57/2043)

[1]Symbols refer to the sex of the gene-drive parents: ♂ male gene-drive parents; ♀ female gene-drive parents.
[2]Drive efficiency was determined by calculating the percentage of progeny expressing the Cherry marker gene out of the total originating from the mating event.
[3]Eye phenotype was determined by calculating the percentage of heteroallelic progeny containing one copy of the drive element (Cherry$^+$) and one resistant mutant *cardinal* (cd^-) allele out of the total originating from the mating event.
[4]Eye phenotype was determined by calculating the percentage of progeny showing the tear phenotype out of the total originating from the mating event.

Table S21. AcTP13 long term cage trial drive efficiency experiment Year 2.

Generation	Male-founder lineage drive efficiency[1,2]		Female-founder lineage drive efficiency[1,2]	
**F_1**	♂ 99.9% (3429/3431)		♀ 99.3% (3266/3289)	
**F_2**	♂ 100% (2886/2886)	♀ 98.4% (2599/2640)	♂ 99.8% (2878/2885)	♀ 97.9% (2742/2801)
**Generation**	**Male-founder lineage *cardinal* eye[3]**		**Female-founder lineage *cardinal* eye[3]**	
**F_1**	♂ 0% (0/3431)		♀ 0.4% (12/3289)	
**F_2**	♂ 0% (0/2886)	♀ 0.08% (4/2640)	♂ 0% (0/2885)	♀ 0.2% (6/2801)
**Generation**	**Male lineage tear eye[4]**		**Female lineage tear eye[4]**	
**F_1**	♂ 0.9% (29/3431)		♀ 10.8% (355/3289)	
**F_2**	♂ 1.5% (44/2886)	♀ 8.1% (213/2640)	♂ 1.6% (45/2885)	♀ 7.9% (220/2801)

[1]Symbols refer to the sex of the gene-drive parents: ♂ male gene-drive parents; ♀ female gene-drive parents.
[2]Drive efficiency was determined by calculating the percentage of progeny expressing the CFP marker gene out of the total originating from the mating event.
[3]Eye phenotype was determined by calculating the percentage of heteroallelic progeny containing one copy of the drive element (CFP^+) and one resistant mutant *cardinal* (*cd^-*) allele out of the total originating from the mating event.
[4]Eye phenotype was determined by calculating the percentage of progeny showing the tear phenotype out of the total originating from the mating event.

Table S22. AcTP43 long term cage trial drive efficiency experiment Year 2.

Generation	Male-founder lineage drive efficiency[1,2]		Female-founder lineage drive efficiency[1,2]	
F_1	♂ 100% (2350/2350)		♀ 99.4% (2536/2551)	
F_2	♂ 100% (2117/2117)	♀ 99.5% (1987/1996)	♂ 97.8% (1983/2028)	♀ 94.1% (1987/2112)
**Generation**	**Male-founder lineage *cardinal* eye[3]**		**Female-founder lineage *cardinal* eye[3]**	
F_1	♂ 0% (0/2350)		♀ 0.5% (14/2551)	
F_2	♂ 0% (0/2117)	♀ 0% (0/1996)	♂ 0% (0/2028)	♀ 0.3% (7/2112)
**Generation**	**Male lineage tear eye[4]**		**Female lineage tear eye[4]**	
F_1	♂ 2.2% (53/2350)		♀ 10.6% (270/2551)	
F_2	♂ 2.1% (45/2117)	♀ 7.4% (147/1996)	♂ 1.6% (32/2028)	♀ 7.0% (148/2112)

[1]Symbols refer to the sex of the gene-drive parents: ♂ male gene-drive parents; ♀ female gene-drive parents.
[2]Drive efficiency was determined by calculating the percentage of progeny expressing the Cherry marker gene out of the total originating from the mating event.
[3]Eye phenotype was determined by calculating the percentage of heteroallelic progeny containing one copy of the drive element (Cherry$^+$) and one resistant mutant *cardinal* (cd^-) allele out of the total originating from the mating event.
[4]Eye phenotype was determined by calculating the percentage of progeny showing the tear phenotype out of the total originating from the mating event.

Table S23. AgTP13 long term cage trial drive efficiency experiment Year 1.

Generation	Male-founder lineage drive efficiency[1,2]		Female-founder lineage drive efficiency[1,2]	
F_1	♂ 100% (2325/2325)		♀ 98.4% (2082/2116)	
F_2	♂ 99.4% (975/981)	♀ 97.5% (955/979)	♂ 98.3% (1068/1087)	♀ 92.5% (1311/1417)
**Generation**	**Male-founder lineage *cardinal* eye[3]**		**Female-founder lineage *cardinal* eye[3]**	
F_1	♂ 0% (0/2325)		♀ 1.3% (27/2116)	
F_2	♂ 0% (0/981)	♀ 0.9% (9/979)	♂ 0% (0/1087)	♀ 1.1% (16/1417)
**Generation**	**Male lineage tear eye[4]**		**Female lineage tear eye[4]**	
F_1	♂ 1.1% (25/2325)		♀ 24.5% (519/2116)	
F_2	♂ 0.6% (6/981)	♀ 10.2% (17/167)	♂ 0.9% (10/1087)	♀ 13.5% (192/1417)

[1]Symbols refer to the sex of the gene-drive parents: ♂ male gene-drive parents; ♀ female gene-drive parents.
[2]Drive efficiency was determined by calculating the percentage of progeny expressing the CFP marker gene out of the total originating from the mating event.
[3]Eye phenotype was determined by calculating the percentage of heteroallelic progeny containing one copy of the drive element (CFP^+) and one resistant mutant *cardinal* (cd^-) allele out of the total originating from the mating event.
[4]Eye phenotype was determined by calculating the percentage of progeny showing the tear phenotype out of the total originating from the mating event.

Table S24. AgTP13 long term cage trial drive efficiency experiment Year 2.

Generation	Male-founder lineage drive efficiency[1,2]		Female-founder lineage drive efficiency[1,2]	
F_1	♂ 100% (1950/1950)		♀ 99.0% (1997/2017)	
F_2	♂ 99.9% (1348/1349)	♀ 97.9% (1568/1601)	♂ 99.8% (1420/1423)	♀ 93.5% (1259/1347)
**Generation**	**Male-founder lineage *cardinal* eye[3]**		**Female-founder lineage *cardinal* eye[3]**	
F_1	♂ 0% (0/1950)		♀ 0.9% (19/2017)	
F_2	♂ 0% (0/1349)	♀ 0.3% (5/1601)	♂ 0% (0/1423)	♀ 0.3% (4/1347)
**Generation**	**Male lineage tear eye[4]**		**Female lineage tear eye[4]**	
F_1	♂ 1.0% (20/1950)		♀ 20.1% (406/2017)	
F_2	♂ 1.0% (13/1349)	♀ 13.1% (209/1601)	♂ 0.7% (10/1423)	♀ 8.8% (118/1347)

[1]Symbols refer to the sex of the gene-drive parents: ♂ male gene-drive parents; ♀ female gene-drive parents.
[2]Drive efficiency was determined by calculating the percentage of progeny expressing the CFP marker gene out of the total originating from the mating event.
[3]Eye phenotype was determined by calculating the percentage of heteroallelic progeny containing one copy of the drive element (CFP^+) and one resistant mutant *cardinal* (*cd^-*) allele out of the total originating from the mating event.
[4]Eye phenotype was determined by calculating the percentage of progeny showing the tear phenotype out of the total originating from the mating event.

Dataset S1 (separate file). Years 1 and 2 raw parasite challenge data for AcTP13, AcTP43 and AgTP13. (XLSX file)

SI References

1. Carballar-Lejarazú, R., et al. Dual effector population modification gene-drive strains of the African malaria mosquitoes, *Anopheles gambiae* and *Anopheles coluzzii*. *Proc. Natl. Acad. Sci. USA* **120,** e2221118120 (2023).

2. Carballar-Lejarazú, R., et al. Compound effector genes suppress malaria parasite infections in gene-drive population modification strains of the African malaria mosquitoes, *Anopheles gambiae* and *Anopheles coluzzii***.** *G3***,** DOI:10.1093/g3journal/jkag014 (2026).

3. Carballar-Lejarazú, R., et al. Next-generation gene drive for population modification of the malaria vector mosquito, *Anopheles gambiae. Proc. Natl. Acad. Sci. USA* **117,** 22805-22814 (2020).

4. Carballar-Lejarazú, R., Pham, T.B., Tushar, T. & James A.A. Mutant allele formation and inheritance during Cas9/guide RNA-mediated gene drive in a population modification mosquito strain for human malaria control. *Genetics* **231,** iyaf176 (2025).

5. Carballar-Lejarazú R., Tushar T., Pham T.B. & James A.A. Cas9-mediated maternal effect and derived resistance alleles in a gene-drive strain of the African malaria vector mosquito, Anopheles gambiae. *Genetics.* **221,** iyac055 (2022).

6.Volohonsky, G., et al. Tools for Anopheles gambiae Transgenesis. *G3* 5, 1151–1163 (2015).

7. Pham, T.B., et al. Experimental population modification of the malaria vector mosquito, *Anopheles stephensi. PLoS Genetics* **15,** e1008440 (2019).

8. Tripathi, A.K., Mlambo, G., Kanatani, S., Sinnis, P. & Dimopoulos, G. *Plasmodium falciparum* Gametocyte Culture and Mosquito Infection Through Artificial Membrane Feeding. *J Vis Exp.* 3, 10.3791/61426 (2020).

9. Dong, Y., Simões, M.L., & Dimopoulos, G. Versatile transgenic multistage effector-gene combinations for *Plasmodium falciparum* suppression in *Anopheles*. *Science advances* **6,,** eaay5898 (2020).
